# Exercise Training Improves Exercise Capacity and Quality of Life in Patients with Inoperable or Residual Chronic Thromboembolic Pulmonary Hypertension

**DOI:** 10.1371/journal.pone.0041603

**Published:** 2012-07-25

**Authors:** Christian Nagel, Felix Prange, Stefan Guth, Jochen Herb, Nicola Ehlken, Christine Fischer, Frank Reichenberger, Stephan Rosenkranz, Hans-Juergen Seyfarth, Eckhard Mayer, Michael Halank, Ekkehard Grünig

**Affiliations:** 1 Centre for Pulmonary Hypertension, Thoraxclinic at University Hospital Heidelberg, Heidelberg, Germany; 2 Department of Thoracic Surgery, Kerckhoff-Klinik Bad Nauheim, Bad Nauheim, Germany; 3 Department of Human Genetics, University of Heidelberg, Heidelberg, Germany; 4 Department of Pneumology, University Gießen-Marburg, Gießen, Germany; 5 Department of Cardiology, University of Cologne, Cologne, Germany; 6 Department of Pneumology, University of Leipzig, Leipzig, Germany; 7 Department of Pneumology, University of Dresden, Dresden, Germany; University of Virginia Health System, United States of America

## Abstract

**Background:**

Aim of this prospective study was to evaluate the effects of exercise training in patients with inoperable or residual chronic thromboembolic pulmonary hypertension (CTEPH).

**Methods:**

Thirty-five consecutive patients with invasively confirmed inoperable or residual CTEPH (16 women;19 men; mean age 61±15 years, mean pulmonary artery pressure, 63±20 mmHg; primary inoperable n = 33, persisting pulmonary hypertension after pulmonary endarterectomy n = 2) on stable disease-targeted medication received exercise training in-hospital for 3 weeks and continued at home for 15 weeks. Medication remained unchanged during the study period. Efficacy parameters have been evaluated at baseline, after 3 and 15 weeks by blinded-observers. Survival rate has been evaluated in a follow-up period of median 36.4 months (interquartile range 26.6–46.6 months).

**Results:**

All patients tolerated exercise training without severe adverse events. Patients significantly improved the mean distance walked in 6 minutes compared to baseline by 61±54 meters after 3 weeks (p<0.001) and by 71±70 meters after 15 weeks (p = 0.001), as well as scores of quality-of-life questionnaire, peak oxygen consumption and maximal workload. NT-proBNP improved significantly after 3 weeks of exercise training (p = 0.046). The 1-year survival rate was 97%, 2-year survival rate was 94% and the 3-year-survival 86% respectively.

**Conclusion:**

Training as add-on to medical therapy may be effective in patients with CTEPH to improve work capacity, quality of life and further prognostic relevant parameters and possibly improves the 1-, 2- and 3-year survival rate. Further multicentric randomized controlled studies are needed to confirm these promising results.

**Trial Registration:**

ClinicalTrials.gov NCT01398345

## Introduction

Chronic thromboembolic pulmonary hypertension (CTEPH) is a rare complication of acute pulmonary embolism due to unresolved emboli initiating remodeling of the non-obstructed pulmonary arteries leading to progressive increase in pulmonary vascular resistance (PVR) [1]. The incidence of CTEPH is not known, but recent studies suggest that 1–4.6% of patients develop the condition within 2 years after an episode of acute pulmonary embolism [2], [3], [4]. Pulmonary endarterectomy (PEA) with removal of the obstructive material is the only curative treatment and can be performed in about 60% of patients [5]. However, about 40% of patients with CTEPH are not operable [5] and 16% to 32% of operated patients suffer from persistent or recurrent pulmonary hypertension (PH) [6]. Clinical presentation with right heart failure and histological damage of pulmonary arteries is similar in CTEPH and pulmonary arterial hypertension (PAH). Therefore, CTEPH patients may benefit from medical therapy that has been approved for PAH. Various uncontrolled clinical trials propound the hypothesis that prostanoids, endothelin receptor antagonists (ERAs) and phosphodiesterase type-5-inhibitors may improve hemodynamics and exercise capacity in patients with operable or inoperable CTEPH [7]. However, the only 2 controlled trials in CTEPH were inconclusive. Subgroup analysis of 57 patients with CTEPH of the Aerosolized Iloprost Randomization (AIR) study showed an improvement in quality of life but failed to prove a significant benefit on exercise capacity and hemodynamics [8]. The BENEFIT-study with Bosentan in CTEPH resulted in a significant improvement of pulmonary vascular resistance and Borg index but did not improve exercise capacity measured by 6-minute walking distance, WHO-functional class (WHO-FC) or time to clinical worsening [9]. Thus, until now beside lifelong anticoagulation no further medical therapy has been approved in Europe or USA for treatment in CTEPH [10], [11]. Selected patients may benefit of PH-targeted drug therapy but cautious use is advised by current guidelines [10], [11]. Preoperative treatment has been reported to have minimal effect on pre-PEA hemodynamics and no effect on post-PEA outcome [12], and may induce unnecessary delay to a potentially curative surgical intervention [12], [13]. Although studies in CTEPH already showed an improvement of survival rate compared to earlier trials without optimized medical PH-targeted therapy [14] survival is still unsatisfying with 82–87%, 75–77% and 70–77% after 1, 2 and 3 years, respectively [15], [16].

Therefore, patients with inoperable or residual CTEPH have a need for additional therapeutic tools addressing their exercise capacity, quality of life and survival. Exercise training has been a useful add-on therapy in other forms of PH [17], [18]. The effect of exercise training in patients with inoperable or residual CTEPH has not yet been evaluated systematically. The objective of the present study was to evaluate safety and effectiveness of exercise training in patients with inoperable or residual CTEPH as add on to optimized medical PH-targeted therapy and to analyze long-term survival.

## Methods

### Study Population and Design

The protocol for this trial and supporting CONSORT checklist with flow-chart are available as supporting information; see Checklist S1, flow-chart Figure S1 and Protocol S1.

Our investigation included patients between 18 and 80 years with CTEPH and WHO-FC II–IV who received exercise and respiratory therapy as add-on to PH -targeted medication between June 2006 and October 2011. Patients had to be stable under optimized medical therapy including inhaled ERAs, inhaled or parenteral prostanoids, phosphodiesterase inhibitors, anticoagulants, diuretics, and supplemental oxygen for least 2 months before entering the study. According to current guidelines [11], [12] all patients underwent a detailed clinical work up at the participating PH centers, including right heart catheterization, ventilation/perfusion lung scan, computed tomography angiography and pulmonary angiography to establish the diagnosis. The status “inoperable CTEPH” had been confirmed by experienced PEA-surgeons (SG, EM). All patients gave written informed consent for this study, which was approved by the Ethics Committee of the University of Heidelberg.

### Outcome Measures

Efficacy parameters were assessed at baseline, week 3 and week 15 as described previously [17], [18]. 6-minute-walking-distance (6MWD) was performed under standardized conditions [19]. Cardiopulmonary exercise testing and stress Doppler echocardiography were carried out during supine bicycle exercise as described previously [17]. Workload, heart rate, systolic pulmonary artery pressure (sPAP), systolic (RRsys) and diastolic (RRdias) systemic blood pressures, ventilation (VE), oxygen uptake (VO_2_), oxygen pulse (VO_2_/heart rate), and carbon dioxide output (VCO_2_) were evaluated continuously. V-slope method was used to detect the anaerobic threshold (AT). We analyzed gas exchange, Borg dyspnea index (with 6 representing no exertion and 20 maximal exertion) [20] and changes in WHO-FC after 3 and 15 weeks. Health related quality of life was measured by the Short Form Health Survey questionnaire (SF-36) [21] at baseline was compared to the results after 15 weeks. Serum N*-*terminal pro brain natriuretic peptide (NT-proBNP) was obtained at baseline and after 3 and 15 weeks. 6MWD, SF-36-questionnaire and NT-proBNP have been obtained and analyzed by investigators who have been blinded to clinical data of the patients.

### Exercise Training Program

We designed a program for patients with PH with a minimum of 1.5 h/day exercise training as described earlier [17], [18], [22]. For the first 3 weeks the program took place in the Rehabilitation Clinic Koenigstuhl Heidelberg. The different parts of the program were interval bicycle ergometer training at low workloads, walking, respiratory training at 5 days/week and dumbbell-training of single muscle groups using low weights (500–1000 g). Patients continued training at home for at least 30 minutes/day at 5 days a week for 12 weeks. Additional to physical training patients received psychological support and performed mental training helping them to improve their perception of individual physical abilities and limits. During the study period the program was closely supervised by physicians specialized in rehabilitation medicine and PH-experts as described earlier [17], [18], [22]. Adverse events were recorded whenever they occurred. Oxygen saturation (sO2) and heart rate were monitored continuously throughout the training and used to adjust the training intensity. When patients’ SO_2_ passed below 90% during exercise they received supplemental oxygen (3–10 L/min) throughout the training. Before leaving the hospital after 3 weeks, patients were given an individualized training manual and a bicycle ergometer for use at home was ordered. Physiotherapists and physicians stayed in close contact to patients and supervised the planned training at home by phone call every 2–4 weeks. All patients were asked to keep close contact to physicians of the training program and to their general practitioners and specialized center.

### Follow-up Assessment

In 2011 all participating patients were interviewed by telephone or at a control visit in the Thoraxklinik Heidelberg using a half-structured questionnaire. The patients were asked for present symptoms, WHO-FC, current medication, what kind of exercise training they pursued at home, for any adverse events of exercise training and any further cardiac events that might have occurred since last observation. If the index patient was deceased, date of death was recorded and their relatives and/or treating physicians were asked for the cause and circumstances of death.

### Statistical Methods

The analyses were performed by a statistician (C.F.). Data are given as mean ± standard deviation and as median, 25% and 75% quantiles for more detailed description at baseline. Statistical tests shown in table 2 were performed without assuming normal distributions. The inner-group comparisons of baseline, weeks 3 and 15 for 6MWD, workload, Borg dyspnea index, parameters of gas exchange, PASP, systemic blood pressure, NT-proBNP, heart rate, summation and subscores of the SF-36 questionnaire were compared by Wilcoxon rank test. WHO-FC comparison at different time points was performed by McNemar-Bowden test. All tests were two sided and p-values <0.05 were considered statistically significant. Bonferroni adjustment for multiple comparisons was performed for comparisons of the primary endpoints such as parameters of quality of life and 6-minute walking distances. In a previous study of 58 patients with pulmonary hypertension of other ethiology we showed an improvement in 6MWD of 84±49 meter. Under a conservative assumption of a difference of 40 meter and a standard deviation of 50 meter, taking multiple testing of 6MWD and 8 quality of life scales by Bonferroni adjustment into account, we calculated (t-test situation) that we would need 30 patients to detect the difference (if present) with a power of 90%. Furthermore, analysis of dropouts that did not attend the 15 week measurement was performed. All analyses were carried out with IBM SPSS V20 (IBM Corp. Armonk, NY, USA). For missing values we performed different imputation strategies and reported the values from the most strict one: 1. multiple imputation using the MCMC method as implemented in SPSS, 2. the last observation carried forward, 3. the baseline carried forward and a pessimistic imputation, in which 3 week 6MWD was imputed as 15 week measurement if it was lower than baseline, otherwise the baseline 6MWD. Kaplan-Meier estimates have been used for survival-analysis with 95% two-sided asymptotic confidence interval (CI). All treated patients were used for the survival analysis. Patients with deaths were counted as endpoints, survivors were regarded as censored.

## Results

### Study Population (Table 1)

We prospectively recruited 39 consecutive patients for this study, 2 had to be excluded due to a change in their PA-targeted medication 2–4 weeks before admission to the rehabilitation hospital, 2 patients had been misdiagnosed as CTEPH and turned out as PAH. Thus, the final study group consisted of 35 consecutive patients in our study group (16 women, 19 men): 33 patients with inoperable CTEPH (94%) and 2 patients with residual CTEPH after PEA (6%). Demographic data, diagnosis, functional class, hemodynamic values, lung function and medical therapy of the study population are summarized in table 1. At baseline 7 patients were in WHO-FC II (20%), 26 patients were classified in WHO-FC III (74%) and 2 in WHO-FC IV (6%). Combination therapies including 2 to 3 PAH-targeted agents were used in 46% of patients (Table 1).

**Table 1:** Baseline Characteristics.

				median	Q25	Q75
Patients [n]		35				
Gender [male/female]	19	/	16			
Age [years]	61	±	15	64	53	71
Height [cm]	170.5	±	10	170	161	178
Weight [kg]	78	±	13	78	70	86
**WHO functional Class – No. [%]**						
II	7		20%			
III	26		74%			
IV	2		6%			
**6MWD [meters]**	408	±	108	420	336	489
**Cardiac Catheterization**						
mPAP [mmHg]	48	±	16	50	35	57
PVR [dyn×sec×cm^−5^]	784	±	399	701	497	945
RA pressure [mmHg]	7.2	±	4.8	7.0	3	10.5
SaO_2_ [%]	91.4	±	3.1	91	90	93.9
PCWP [mmHg]	9.3	±	4.3	8.5	7	12
CI [L/min/m^2^]	2.3	±	0.5	2.3	1.85	2.36
**Echocardiography**						
sPAP at rest, mmHg	64	±	20	60	50	80
sPAP during exercise, mmHg	94	±	25	95	75	110
RV area, cm^2^	28.4	±	10.4	28.5	19	34
RA area, cm^2^	26.0	±	8.5	26.5	18.5	33.5
TAPSE, cm	1.98	±	0.32	1.95	1.8	2.2
**PAH-targeted medication**						
Endothelin Receptor Antagonist	21		60%			
Phosphodiesterase-5-Inhibitor	21		60%			
Prostanoids inhaled	6		17%			
Prostanoids intravenous	1		3%			
Calcium Channel Blockers	2		6%			
Soluble guanyl cyclase Stimulator	2		6%			
**Combination therapy**						
Monotherapy	17		49%			
Dualtherapy	10		29%			
Tripletherapy	6		17%			

Values are given as mean±SD, median and 25 and 75% Quantile (Q25, Q75) or as n and %. mPAP  =  mean pulmonary arterial pressure, PVR  =  pulmonary vascular resistance, RA =  right atrium, SaO_2_ =  oxygen saturation, PCWP  =  pulmonary capillary wedge pressure, CI =  Cardiac Index, sPAP  =  systolic pulmonary arterial pressure, RV =  right ventricle, TAPSE  =  tricuspid plane annular systolic excursion.

### Assessment of Training Effects

**Figure 1:**
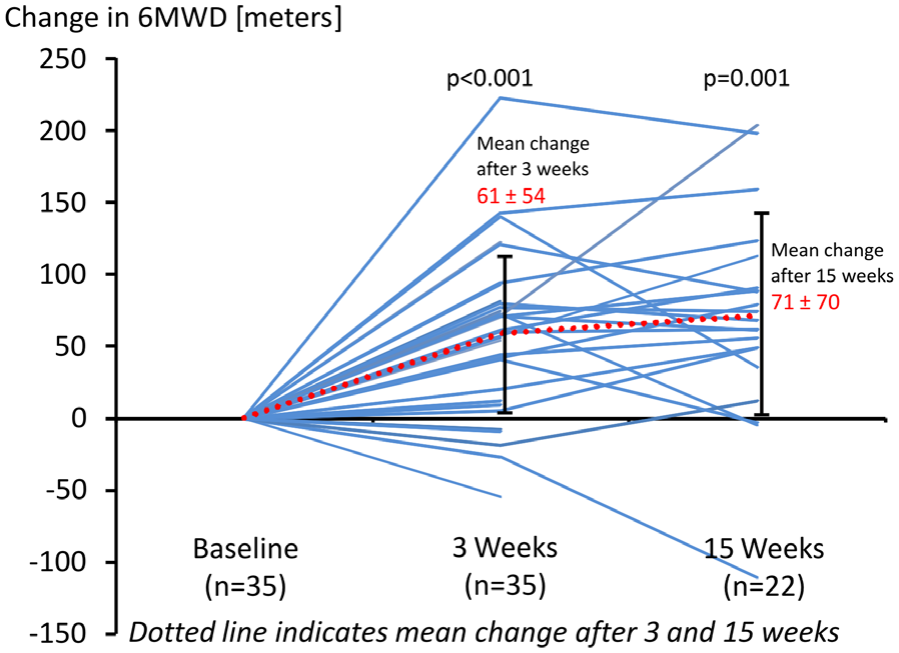
Individual changes in Six-Minute-Walking Distance (6MWD) after 3 and 15 weeks exercise training. With the use of Wilcoxon Rank Test according to baseline walking distance, p<0.001 was obtained for the comparison to baseline with weeks 3 (n = 35) and p = 0.001 with week 15 (n = 22). The dotted line indicates the mean change from baseline in 6MWD (61±54 meters and 71±70 meters).

Training significantly improved the 6MWD from 408±108 meters by 61±54 meters after 3 weeks (p<0.001) and by 71±70 meters after 15 weeks (p = 0.001) (Figure 1, Table 2). All patients except six improved in 6MWD (Figure 1). The patient with the highest decrease of 6MWD had experienced a syncope as described in the adverse events section and walked intentionally less after 3 and 15 weeks in order to avoid another syncope. Three of the 6 further “non-responders” deceased during follow-up (after 2, 2.2 and 2.8 years, respectively). In one of these patients other parameters of physical exercise capacity had improved during training. Two further patients improved in their 6MWD after 3 weeks but had a slight decrease compared to baseline after 15 weeks. All other patients improved in their 6MWD, most of them continued with exercise training at home.

**Table 2:** Efficacy parameters.

Characteristic	Baseline (n = 35)				3 weeks (n = 35)			p-value			15 weeks (n = 22)									p-value		
**6MWD**																						
Walking distance [meters]	408	±	108		468	±	130						509	±			81					
mean change [meters]					60.5	±	54	<0.001	*				71	±			70	0.001		*		
95% CI for the difference to baseline				41.3		–	79.8					39.2			–	102.9						
change: median Q25,Q75 [meters]					71.0		41.5	87.0				68.0				42.0		102.0				
**Quality of life Questionnaire SF-36**																						
Physical functioning	36.2	±	20.4								40.9			±			27,4	0.041		#		
Role-physical	33.9	±	36.8								40.9			±			44.0	0.67				
Bodily pain	70	±	28								73.2			±			30.8	0.367				
General health perception	40.9	±	16.6								44.7			±			15.3	0.777				
Vitality	46.4	±	16.6								51.1			±			18.1	0.03		#		
Social functioning	62.9	±	31.9								67.9			±			29.4	0.114				
Role-emotional	54	±	47.5								60.8			±			45.7	0.177				
Mental Health	63.2	±	18.7								64.5			±			21.2	0.174				
**Cardiopulmonary exercise testing**																						
peak VO_2_/kg [mL/Min/kg]	12.1	±	1.7		13.4	±	3.7	0.003	*	14				±			2.9	0.017		#		
peak VO_2_ [mL/min]	933.2	±	335.3		1028.8	±	330.6	0.022	#	1111				±			304.2	0.014		#		
EqCO_2_ at AT [mL/min]	50	±	10.9		47.6	±	11.7	0.264		48				±			12.8	0.953				
VO_2_ at AT [mL/min]	672.8	±	236.4		638	±	242.3	0.872		846.4				±			259.7	0.441				
O_2_-pulse [(mL/min)/min-1]	8.2	±	2.7		8.8	±	2.9	0.345		8.8				±			3.1	0.185				
HR rest [min-1]	73.2	±	12		70.8	±	10.2	0.657		73.8				±			11.8	0.679				
HR max [min-1]	114.8	±	19.7		118.9	±	23.5	0.049	#	129.5				±			19.4	0.010		#		
RR sys rest [mmHg]	117.6	±	15.5		118.4	±	13.4	0.989		112.8				±			30.3	0.852				
RR dia rest [mmHg]	77.5	±	11.3		76.8	±	9.1	0.266		78.3				±			9.9	0.924				
RR sys max [mmHg]	143.5	±	27.5		150.1	±	26	0.210		143.2				±			43.8	0.586				
RR dia max [mmHg]	85.4	±	15		83.1	±	20.4	0.653		89.6				±			13.6	0.811				
Oxygen saturation rest [%]	93	±	6.2		92.8	±	6.6	0.472		95.2				±			2.2	0.605				
Oxygen saturation max [%]	87.9	±	7.8		88.7	±	6.8	0.820		89.9				±			3.5	0.440				
sPAP rest [mmHg]	63.5	±	20.3		62.5	±	20.5	0.710		56.8				±			15.9	0.083				
sPAP max [mmHg]	94.1	±	25.2		91.9	±	32.9	0.458		94.1				±			27	0.243				
Workload max [Watt]	64.1	±	28		76.7	±	27.8	0.005	*	90				±			22	0.010		#		
Borg Scale	14.9	±	2.3		15.6	±	2.2	0.071		16.2				±			1.6	0.015		#		
**Laboratory parameters**																						
NT-proBNP [pg/mL]	2334.5	±	2886.8		1715.9	±	2263.4	0.046	#	2500.6				±			2896.7		0.339			

Values are mean±SD, q25∶25% quantile, q75∶75% quantile, CI = 95% confidence interval 6MWD,

∼p<0.08; #p<0.05; *p<0.01 in comparison to baseline we show Wilcoxon test p-values.

6-MWD  = 6-minute walking distance, VO2/kg  =  max.oxygen consumption/kg, HR  =  heart rate, RR  =  Blood pressure.

sPAP  =  systolic Pulmonary arterial pressure.

**Figure 2:**
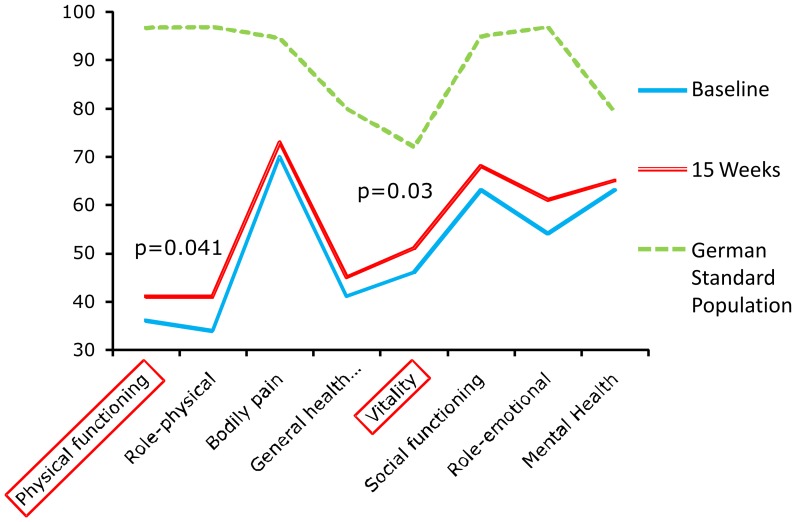
Mean SF-36 scores of Quality of life Subscales (SF-36 questionnaire) before and after Exercise Training. At baseline (straight line), mean SF-36 scores were significantly reduced in comparison to respective values of a normal population (dotted line). After 15 weeks (dashed line), the 2 subscales of the SF-36 questionnaire physical functioning and vitality improved significantly. P-values are indicated vs. baseline. No significant improvement was found for role emotional (ROLEM), role physical (ROLPH), general health (GH), social functioning (SF), mental health (MH), bodily pain (PAIN) after training. With Bonferroni adjustment, values of p<0.005 preserve statistical significance. At baseline data of 28 patients, after 15 weeks of 23 patients were available and included.

Training also significantly improved quality of life parameters indicated by the SF-36 subscale scores for physical functioning (p = 0.041) and vitality (p = 0.03, Figure 2, Table 2). Using Bonferroni adjustment for 6MWD and quality of life scales improvement of 6 MWD remained statistically significant.

Mean peak oxygen consumption, peak oxygen consumption per kg body weight, workloads with an increase of maximal heart rate during cardiopulmonary exercise testing increased significantly from baseline to 3 weeks and to 15 weeks (Table 2). NT-proBNP plasma levels were significantly reduced after 3 weeks. The change in WHO-FC compared to baseline after 3 weeks and after 15 weeks was not significant (p = 0.157 and p = 0.157).

### Missing Value Analysis

Thirteen patients (37%) did not attend the visit after 15 weeks (12 referred from other PH-centers than Heidelberg) mainly due to the long travel distance. They showed at baseline no significant differences for most parameters except maximal systolic and diastolic blood pressure. However, although statistically not significant, patients had a tendency of being older, having a higher BMI, lower 6MWD at baseline and after 3 weeks and lower improvement of 6MWD and slightly worse values for all quality of life scales in comparison to patients who completed all visits.

Results remained significant after imputation of missing values for the 6 MWD at 15 weeks using the rules described in the methods: a) the last observation carried forward method revealed an improvement of 60.2±62.5 Meters, p<0.001; b) the baseline carried forward revealed 42.6±62.5 meters, p<0.001; c) pessimistic imputation: improvement of 40.8±66.1 meters, p = 0.001.

### Adverse Events

During the 3 weeks in-hospital training 5 patients had an adverse event, in two cases (5.7%) it was related or possibly related to the training program. In one patient syncope occurred during the in-hospital rehabilitation after climbing three flights of stairs. Intensity of exercise training was reduced and the patient was able to continue the program without any further events. He was however concerned to get another syncope and walked intentionally less in the test of 6MWD after 3 and 15 weeks. The other patient had a herpes zoster infection 2 months after in-hospital training, which was possibly related to exercise training and was treated successfully by antiviral therapy and continued afterwards. In the other 3 patients adverse events had been respiratory infections, which were not related to the training itself. These patients had to interrupt the training program during the in-hospital rehabilitation and were treated successfully by antibiotic therapy. They continued with the training after recovery and showed an improvement in 6MWD and peak oxygen consumption after 15 weeks. All other patients (86%) tolerated exercise training well. There were no signs of clinical worsening of right heart failure during the in-hospital program. All patients repor**t**ed that they had improved their awareness of their physical abilities and limitations.

### Follow-up and Survival

**Figure 3:**
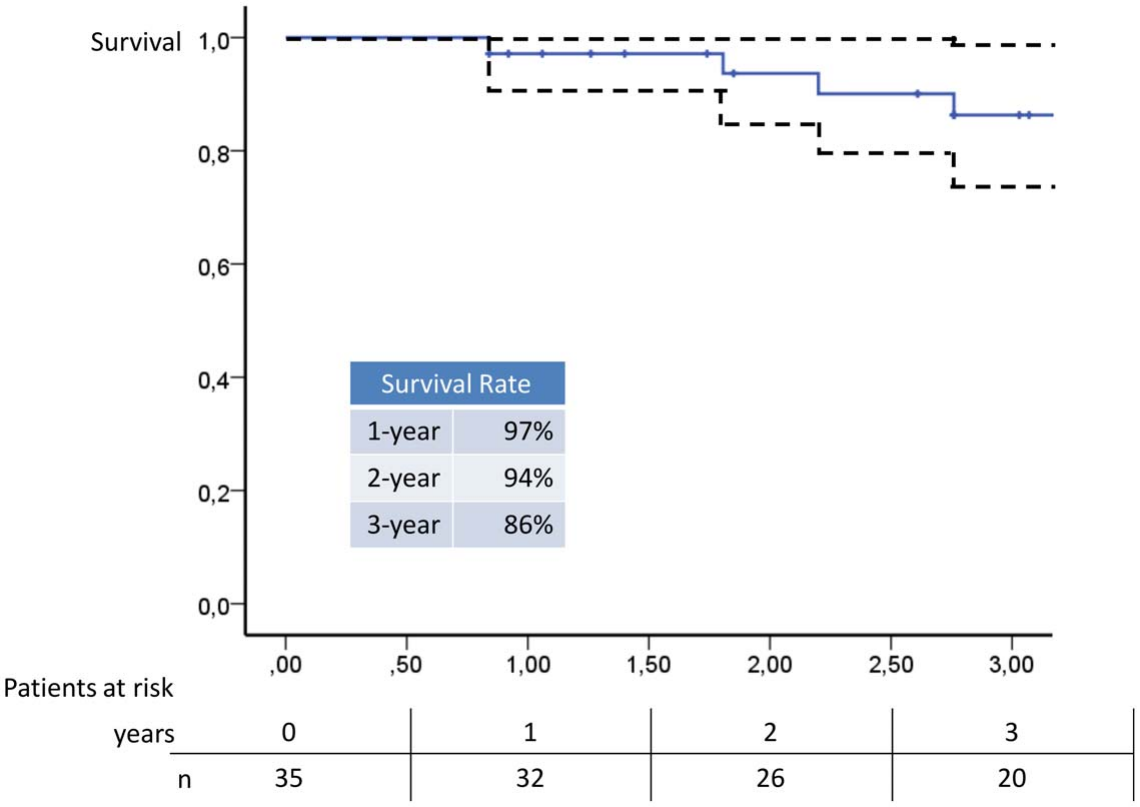
Survival by Kaplan Meier Analysis. Within a follow-up period of median 36.4 months (interquartile range 26.6–46.6 months) 6 patients deceased, 4 due to PH. One patient died due to lung cancer >4 years after baseline. In one patient cause of death remained unknown. The straight line indicates overall survival, with 97% after 1-year, 94% after 2-years and 86% after 3 years. The dashed line indicates 95% Confidence interval.

Follow-up data were obtained after a median of 36.4 months (0.8 to 5.2 years after baseline/start of the in-hospital rehabilitation, interquartile range 26.6–46.6 months). During follow-up 6 patients died. Three due to CTEPH and right heart failure, one patient due to sudden cardiac death, one patient died due to lung cancer 4.2 years after baseline. In one further patient the cause of death remained unknown. The Kaplan-Meier overall-survival rate was 97% after 1-year, 94% after 2-years and 86% after 3 years (figure 3). We interviewed the 29 surviving patients for actual symptoms, worsening events and continuation of exercise training. Of the 29 patients assessed during follow-up, 23 (79%) had been continuing exercise training, 19 for more than 3 years after baseline. 20 of the 23 continued bicycle ergometer training, 13 dumbbell-training, 9 walking, 15 respiratory training and 17 alternative training such as gymnastics. Nine patients combined two items, 5 patients three items and 5 patients four items. Four patients (14%) did not continue the training after the end of the study for the following reasons: one patient discontinued due to clinical worsening, two further patients mentioned lack of time, the fourth patient was mainly limited by musculoskeletal pain and did not see an improvement by exercise training.

## Discussion

This is the first prospective clinical trial investigating short- and long-term effects of exercise training as add-on to PAH-targeted medication in patients with severe CTEPH. The results of the study suggest that training in pulmonary hypertension can significantly improve prognostic relevant parameters as exercise capacity and quality of life in this condition and has an excellent long-term survival of 97% after 1 year, 94% after 2 years and 86% after 3 years, respectively.

The results represent an important source of data on survival, exercise capacity and quality of life in patients with CTEPH treated with exercise and respiratory therapy. Mean 6MWD significantly improved by almost 20%, mean peak oxygen consumption by 16%, and mean maximal workload by 40% during 15 weeks of the training program. This had a significant impact on quality of life improving two of eight scores of the SF36 questionnaire. The positive effect of exercise training was also documented in a significant decrease (>20%) of mean NT-proBNP after 3 weeks. Overall compliance was excellent with a continuation in 79% during the follow-up period of 36.5±17 months. However, these data must be confirmed in randomized, controlled studies. The results of this study are in line with previous studies of training in patients with other forms of PH/PAH [17], [18], [22], [23], [24]. These studies showed similar effects in patients with various forms of PH and right heart insufficiency in a randomized controlled study [17] and in single-arm, non-controlled designs [18], [22], [23]. However, in these studies only few patients with CTEPH had been included. Thus, there are almost no previous data on the effect of exercise training in patients with inoperable or residual CTEPH. The promising results of this study suggest that training may be an important add-on therapy in CTEPH regarding long-term survival, exercise capacity, quality of life and oxygen consumption.

### Improvement in 6-Minute-walking-distance

The 6MWD has been used as primary end-point in many randomized controlled clinical trials in PAH [11] and correlated with mortality and prognostically relevant parameters [25]. In patients with medically treated inoperable CTEPH 6MWD was the only independent predictor of long-term survival [16], [26]. Furthermore, 6MWD and NT-proBNP were independent predictors of perioperative mortality [27]. Pre-operative cut-off values for NT-proBNP of 1200 pg/ml and 345 m for 6MWD had both high negative predictive values for mortality [27].

Previous uncontrolled drug-trials with Riociguat [28] and Bosentan [29], [30], [31], [32] in small cohorts of CTEPH-patients showed a significant increase in 6MWD, whereas in a randomized controlled trial Bosentan did not significantly improve 6MWD [10]. Therefore, a mean increase of 61 meters after 3 weeks and 71 meters after 15 weeks exercise and respiratory therapy is unexpectedly high. The absence of a non-trained “placebo” group may be considered a limitation of the study, with concern that some of the improvements were due to “placebo” effect, rather than efficacy of the training program. However, previously reported placebo controlled PAH studies of Bosentan or other PAH-targeted drugs have not shown any clinically relevant improvements in placebo groups. In fact, they have generally shown a decline. Despite the limitations of this study the magnitude of the mean improvements from baseline in 6MWD and other parameters as quality of life makes the findings promising. Further randomized, controlled studies are warranted.

### Survival of Patients with CTEPH after Training

The survival estimate of 97% after 1 year, 94% after 2 years and 86% after 3 years using exercise training as add-on to optimized PAH targeted medical treatment can be considered as a positive outcome in CTEPH. Despite optimized PAH-targeted therapy several studies report a lower 1 year (87%/82%), 2 year (77%/75%) and 3 year survival rate (77%/70%) [15], [16]. One study showed a comparable 1 year survival rate of 96% [31].

The CTEPH-cohort assessed in this study has been severely affected with a mean 6MWD of 408±108 meters at baseline despite double or even triple PAH targeted therapy in 46% of patients and is therefore comparable to the cohorts described previously. However, we cannot exclude that we selected highly motivated and compliant patients with less outcome limitations. Nevertheless, exercise training may have improved survival by ameliorating prognostic relevant factors as quality of life and exercise capacity. Since maximal heart rate during cardiopulmonary exercise testing and peak oxygen consumption significantly improved, exercise training possibly improved right ventricular contractile reserve [18]. Similar effects of training have been seen in patients with IPAH and right heart failure [18], [22].

### Limitations

Limitations of our study include its relatively small sample size and lack of randomization. Because of this, there could be a referral bias that CTEPH patients who do well have been selected. Furthermore patients were well-aware of the assignment to a training study, which may also have favored the results. The effects of exercise training after 15 weeks may be further biased due to the missing values of about 37% of patients who did not perform the last follow-up visit. However, the efficacy after 3 weeks exercise and respiratory therapy and the high proportion of patients who continued with the program suggest a positive effect in this cohort. The significant effect and high compliance of exercise training in inoperable CTEPH might also be due to the closely supervised in-hospital rehabilitation program, which probably cannot be simply translated to an out-patient program. This limits the application of this program in other countries which cannot provide in-hospital rehabilitation care. Therefore, further studies are necessary using ambulatory training programs. It is a general issue of rehabilitation programs that the therapy cannot be performed in a blinded fashion and that a referral bias towards highly motivated patients with a better outcome may occur. Further studies are necessary to determine the effects of training programs on outcome in patients with pulmonary hypertension.

### Conclusions

This is the first trial investigating exercise training in CTEPH as add-on to optimized medical therapy. The results indicate that exercise training is effective in CTEPH and may improve work capacity, quality of life and further prognostic relevant parameters. Exercise and respiratory therapy possibly improves survival rate. Further randomized studies are needed to confirm these promising results.

## Supporting Information

Figure S1 CONSORT Flow-chart
(TIF)

Checklist S1 CONSORT Checklist
(DOC)

Protocol S1 Trial Protocol
(DOC)
