# Supplementary figures and images for: Exercise Training Improves Exercise Capacity and Quality of Life in Patients with Inoperable or Residual Chronic Thromboembolic Pulmonary Hypertension
Source: PLoS One. 2012 Jul 25;7(7):e41603. doi: 10.1371/journal.pone.0041603 (PMC3404995; doi:10.1371/journal.pone.0041603)

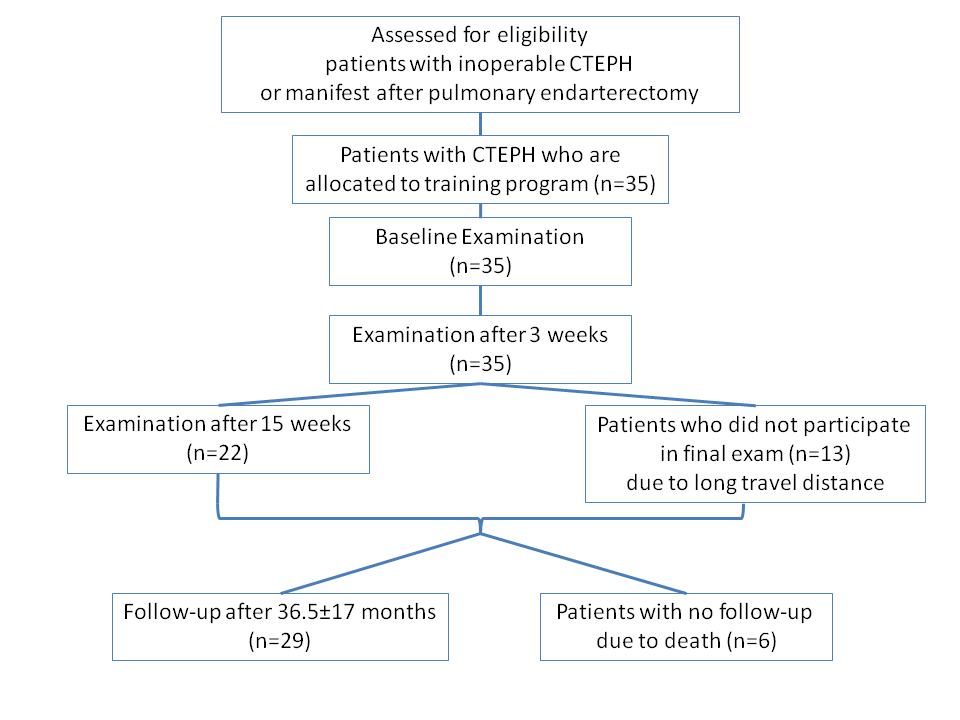

Supplement: Figure S1 — CONSORT Flow-chart (TIF) [file pone.0041603.s001.tif]
