# Supplementary material for: Exercise Training Improves Exercise Capacity and Quality of Life in Patients with Inoperable or Residual Chronic Thromboembolic Pulmonary Hypertension
Source: PLoS One. 2012 Jul 25;7(7):e41603. doi: 10.1371/journal.pone.0041603 (PMC3404995; doi:10.1371/journal.pone.0041603)
Supplement: Protocol S1 — Trial Protocol (DOC) [file pone.0041603.s003.doc]

**Atemtherapie und Bewegungstherapie bei chronischer pulmonaler Hypertonie**

**Leiter der Studie/**

**verantwortlicher Studienarzt:** PD Dr. med. Ekkehard Grünig

Universitätsklinikum Heidelberg

Innere Medizin III, Kardiologie

Bergheimer Str. 58

69115 Heidelberg

Tel.: 06221- 56 8906

Piepser: 06221- 56 38689

Fax: 06221- 56 5447

E-Mail: [ekkehard_gruenig@med.uni-heidelberg.de](mailto:ekkehard_gruenig@med.uni-heidelberg.de)

**Initiator, Koordinator und Schirmherr der Studie:** Bruno Kopp, Pulmonale Hypertonie e.V. Wormser Str. 20, 76287 Rheinstetten, Tel. 07242/952666, Fax: 952667

**Weitere Beteiligte:**

Prof. Dr. med. J. Buß und Frau Dr. med. Karger PD Dr. med. Heinrike Wilkens

Rehabilitationsklinik Königstuhl Universitätskliniken des Saarlandes

Kohlhof 8 Innere Medizin V

69117 Heidelberg Kirrberger Straße, Gebäude 91

Tel.: 06221- 90 7501 Homburg/Saar

E-Mail: [koenigstuhl@gmx.de](mailto:koenigstuhl@gmx.de) Tel.: 06841- 16-23619 oder -23600

[Gabriele.Karger@lva-bw.de](mailto:Gabriele.Karger@lva-bw.de) E-Mail: [inhwil@med-rz.uni-sb.de](mailto:inhwil@med-rz.uni-sb.de)

[jan.buss@rehaklinik-koenigstuhl.de](mailto:jan.buss@rehaklinik-koenigstuhl.de)

Frau Dr. med. Jana Jünger Dr. med. Sebastian Ley, Dr. Christian Fink

Universitätsklinikum Heidelberg DKFZ Heidelberg

Innere Medizin II Abteilung für onkologische

Bergheimer Str. 58 Diagnostik und Therapie

69115 Heidelberg Im Neuenheimer Feld 280

Tel.:06221- 56 8246 oder 56 8657 69120 Heidelberg

Piepser: 06221- 56 38872 Tel.: 06221-42 2564 oder 42 2608

Fax: 06221- 56 1341 Fax: 06221 42 2567

E-Mail: [jana_juenger@med.uni-heidelberg.de](mailto:jana_juenger@med.uni-heidelberg.de) E-Mail: [ley@gmx.de](mailto:ley@gmx.de)¸ [c.fink@dkfz.de](mailto:c.fink@dkfz.de)

Sandra Kreuscher / Dr. med. Derliz Mereles Dr. med. Michael Halank

Universitätsklinikum Heidelberg Universitätsklinikum Dresden

Innere Medizin III, Kardiologie Innere Medizin I, Pneumologie

Bergheimer Str. 58 Fetscher Str. 74

69115 Heidelberg 01307 Dresden

Tel.: 06221- 56 8906 Tel. 0351-458-3345 oder -4580

E-Mail: [derliz_mereles@med.uni-heidelberg.de](mailto:derliz_mereles@med.uni-heidelberg.de) [michael.halank@uniklinikum-dresden.de](mailto:michael.halank@uniklinikum-dresden.de)

[sandra_kreuscher@med.uni-heidelberg.de](mailto:sandra_kreuscher@med.uni-heidelberg.de)

PD Dr. med. Ingram Schulze-Neick PD. Dr. Olschewski, Dr.Stefanie Ghofrani

Deutsches Herzzentrum Berlin Pulmonale Hypertonie Ambulanz

Abteilung für angeb.Herzfehler/Kinderkardiologie Innere Med. II, Uniklinik Giessen

13353 Berlin Klinikstrasse 36

Tel. 030/4593/2890 oder -2820 35392 Giessen

Fax: 030/4593/2900 Tel: 0641/994-2534/5 Fax: 2519

mail: [schulze-neick@dhzb.de](mailto:schulze-neick@dhzb.de) [stefanie.ghofrani@innere.med.uni-giessen.de](mailto:stefanie.ghofrani@innere.med.uni-giessen.de) [Horst.Olschewski@innere.med.uni-giessen.de](mailto:Horst.Olschewski@innere.med.uni-giessen.de)

Dr. Phil. Marc Immenroth Prof. Dr. med. Marius Höper

Dr. Jan Mayer Medizinische Hochschule Hannover
Dipl. Psych./Sportwissenschaftler Abteilung Pneumologie
Institut für Sport und Sportwissenschaft Carl-Neuberg-Str. 1
INF 720 30625 Hannover
69120 Heidelberg Tel. +49-511-532-3536
Tel. 06221/54-4646 Fax. + 49-511-532-8536
Handy: 0172/7329535; E-mail [hoeper.marius@mh-hannover.de](mailto:hoeper.marius@mh-hannover.de)

Handy Mayer:0171/7567761

mail: [mimmenro@ix.urz.uni-heidelberg.de](mailto:mimmenro@ix.urz.uni-heidelberg.de)

[jmayer@ix.urz.uni-heidelberg.de](mailto:jmayer@ix.urz.uni-heidelberg.de)

PD Dr. med. Felix Herth

Thoraxklinik am Universitätsklinikum Heidelberg

Amalienstr. 5

69126 Heidelberg

Tel.06221/396-0; -600

Fax:06221/396-802

Mail: [felix.herth@thoraxklinik-heidelberg.de](mailto:felix.herth@thoraxklinik-heidelberg.de)

[f@herth.net](mailto:f@herth.net)

**Datum:** 26.11.2003 Geplante Projektdauer: 2 Jahre

# Zusammenfassung

Die schwere chronische pulmonale Hypertonie (PH) ist eine Erkrankung, die zu einer progredienten Rechtsherzbelastung führt und mit einer starken Einschränkung der körperlichen Leitungsfähigkeit, der Lebensqualität und der Überlebensrate einhergeht. Im amerikanischen Register für PH betrug die mittlere Überlebensrate lediglich 2.8 Jahre. In aktuellen, randomisierten Studien wurde bei Patienten mit PH und NYHA-Klasse III-IV eine Verbesserung der 6-Minuten-Gehstrecke um im Mittel 30 - 50 Meter sowie eine Zunahme der Lebensqualität durch die Therapie mit inhalativem Prostazyklin und Endothelin-1-Antagonisten nachgewiesen. Als unterstützende Maßnahme wird den Patienten mit chronischem Lungenhochdruck in der Regel die strikte körperliche Schonung empfohlen. Es ist bislang aber nicht untersucht, ob dies wirklich hilfreich ist. Bislang gibt es keine speziellen Rehabilitations-Maßnahmen für Patienten mit PH. Sowohl bei Gesunden, als auch bei einer Vielzahl von Erkrankungen, insbesondere bei der chronischen Linksherzinsuffizienz (Hambrecht 2000, 2002) und der koronaren Herzerkrankung (Schuler 2002) wurde der positive Einfluss regelmäßiger körperlicher Aktivität auf die Prognose, die Leistungsfähigkeit, das subjektive Wohlbefinden, die oxidative Kapazität der Skelettmuskulatur, die Endothelfunktion sowie die Ventilation nachgewiesen.

Ziel der vorliegenden Pilotstudie ist, bei Patienten mit PH in NYHA- Klasse II-III, die seit 3 Monaten unter maximal optimierter medikamentöser Therapie stabil sind, die Wirkung einer individuell abgestimmten, vorsichtigem Atem- und Bewegungstherapie auf die 6-Minuten-Gehstrecke, die maximale Sauerstoffaufnahme (VO2 max.), das Befinden, die Hämodynamik sowie die Lebensqualität zu untersuchen. Dabei sollen die Studienteilnehmer in 2 Gruppen randomisiert werden: eine Gruppe A, bei der die Patienten eine konventionelle 3-wöchige Kur erhalten, in der körperliche Anstrengung vermieden und gesunde Ernährung, Massagen, Fortbildung, Unterhaltung, Erholung im Vordergrund stehen soll (Urlaubscharakter). Die Teilnehmer in der Gruppe B sollen zusätzlich eine Atem- und Bewegungstherapie sowie mentales Gehtraining erhalten. Um dabei eine enge Betreuung und Überwachung der Patienten zu gewährleisten, soll die Atem- und Bewegungstherapie auch bei Gruppe B im Rahmen eines 3 wöchigen Kuraufenthaltes in der Rehabilitations-Klinik Königstuhl begonnen und vom Patienten dann über mindestens 3 Monate ambulant fortgeführt werden. Die körperlichen Belastungen sollen dabei nicht die vom Patient vor der Kur im Alltag vorgenommenen Tätigkeiten übersteigen. Während der Rehabilitationsmaßnahme soll die medikamentöse Therapie beibehalten werden. Die Rehabilitationsmaßnahmen umfassen dabei folgendes Trainingsangebot: Täglich ca. 30 Minuten Atemtherapie, einmal täglich ca. 15 Minuten Intervalltraining auf einem Ergometer (mit 0-30 Watt), isometrische Kraftübungen einzelner Muskelgruppen mit geringen Gewichten (500g), sowie begleitete Spaziergänge auf ebener Erde. Drei mal pro Woche soll zudem ein "mentales Gehtraining" vorgenommen werden, bei dem Bewegungsabläufe gedanklich durchgeführt werden. Zudem sollen durch Videoaufzeichnungen die Bewegungsabläufe weiter analysiert und optimiert werden. Der Trainingspuls und die Trainingsintensität soll nach 3 Monaten kontrolliert und die Trainingseinheiten dann ggf. angepasst werden. Eine rasche Steigerung der Trainingsintensität soll aber auch dann unterbleiben, wenn sie vom Patient gewünscht wird. Zudem wird den Patienten in beiden Gruppen Gespräche mit einem Psychologen angeboten.

Alle Patienten sollen vor Beginn der Rehabilitationsmaßnahme, unmittelbar danach sowie nach 3 Monaten klinisch untersucht werden. Die Kontrolluntersuchungen umfassen die Anamnese, körperliche Untersuchung, EKG und Echokardiographie in Ruhe und unter Belastung, die Spiroergometrie sowie eine Magnetresonanztomographie.

# Inhaltsverzeichnis:

# 1. Einleitung

- 1. Wissenschaftliche Grundlagen
  2. Eigene Vorarbeiten

**2. Ziele der Studie/Zielkriterien**

**3. Zu prüfendes Verfahren (nur soweit einschlägig)**

# 4. Studiendesign(-typ)

# 5. Randomisierungsverfahren/ggf. -plan

# 6. Einschlusskriterien

**7. Ausschlusskriterien**

**8. Studienablauf**

**9. Therapieelemente im Rahmen der stationären Rehabilitationsmaßnahme:**

9.1. Atemtherapie

- 1. Ergometerintervalltraining
  2. Begleitete Spaziergänge
  3. leichtes Muskelaufbautraining einzelner Muskelgruppen
  4. psychologische Gespräche
  5. progressive Muskelentspannung
  6. mentales Gehtraining
  7. weitere Maßnahmen/Angebote

**10. Kontrollparameter (siehe Begleitbogen CRF)**

**11. Begleittherapie**

# 12. Abbruchkriterien

**13. Ethische und rechtliche Aspekte**

## Ethische Grundlagen

- 1. Patienten-/Probandeninformation/Einverständniserklärung

**14. Rechtliche Grundlagen**

- 1. Votum der Ethikkommission
  2. Datenschutz/Einblick in Originalkrankenunterlagen

**15. Unterschriften**

# Appendices:

1. Patienteneinverständniserklärung

2. Patienteninformation

3. Atemtherapie, Beschreibung der Maßnahmen

4. CRF

5. SF 34

6. 6-Minuten-Gehtest

7. Therapiepläne

# 1. Einleitung

**1.1. Wissenschaftliche Grundlagen**

Eine pulmonale Hypertonie besteht, wenn der pulmonalarterielle Mitteldruck (PAP) in Ruhe 25 mmHg überschreitet (Rich 1987). Für die Symptomatik und die Prognose der Patienten mit PH ist die Einschränkung der Kreislaufreserve entscheidend, die sich aus der Höhe des pulmonalen Perfusionswiderstandes und der rechtsventrikulären Adaptation ergibt. Eine schwere PH ist durch eine Verminderung des Herzzeitvolumens und massiv erhöhte pulmonalarterielle Drucke (PAP >40 mmHg) in Ruhe charakterisiert (Seeger 2001) und geht mit einer erhöhten Nachlast des rechten Herzens mit nachfolgendem Cor pulmonale einher. Die Patienten mit schwerer, chronischer PH haben eine deutlich eingeschränkte körperliche Belastbarkeit und Lebenserwartung (Runo 2003). Im amerikanischen Register für PH betrug die mittlere Überlebensrate lediglich 2.8 Jahre (Rich 1987). Unterschiedliche Pathomechanismen können zur Entwicklung einer PH beitragen und führten in der Weltkonferenz in Evian 1998 sowie in Venedig 2003 zu einer diagnostischen Klassifikation in 5 Gruppen, die "Pulmonal arterielle Hypertonie", „Pulmonal venöse Hypertonie“, den „Erkrankungen der Atemwege/Hypoxie“, die „chronisch thromboembolische pulmonale Hypertonie“ sowie "seltene Krankheiten mit direktem Befall der Lungengefäße". Die Diagnose der PH ist letztlich exakt nur mit dem Rechtsherzkatheter möglich, obwohl der systolische PA-Druck sehr valide auch mittels Echokardiographie bestimmt werden kann. Im Rahmen der Rechtsherzkatheteruntersuchung wird eine Reagibilitätsprüfung vorgenommen, die für die weitere medikamentöse Therapie von entscheidender Bedeutung ist. Zudem muss eine Kaskade von klinischen Untersuchungen vorgenommen werden, einschließlich der Echokardiographie, Lungenfunktionsprüfung, Blutgasanalyse, Röntgen-Thorax, Perfusions-Ventilationsszintigraphie, Linksherzkatheter, Immunologie, Hepatitis-Diagnostik, HIV-Serologie, um eine korrekte Diagnose und Klassifikation vornehmen zu können (Runo 2003). Die schwere, chronische PH ist derzeit noch nicht kausal zu behandeln und kann nur in seltenen Fällen geheilt werden. In aktuellen, randomisierten Studien wurde bei Patienten mit PH und NYHA-Klasse III-IV eine Verbesserung der 6-Minuten-Gehstrecke um im Mittel 30 - 50 Meter sowie eine Zunahme der Lebensqualität durch die Therapie mit inhalativem Prostazyklin und Endothelin-1-Antagonisten nachgewiesen (Runo 2003). Als unterstützende Maßnahme wird den Patienten mit chronischem Lungenhochdruck die strikte körperliche Schonung empfohlen (Seeger 2001). Es ist bislang aber nicht untersucht, ob dies wirklich hilfreich ist. Bislang gibt es keine speziellen Rehabilitations-Maßnahmen für Patienten mit PH. Dabei haben bei den letzten beiden Treffen der Selbsthilfegruppe PHeV einige Patienten berichtet, daß Ihnen körperliche Bewegung, die sie oft entgegen ärztlichen Rat durchführen, gut tut und ihr Befinden sogar verbessert habe. Für Patienten mit chronischem Lungenhochdruck und Rechtsherzbelastung liegen bislang keine Studien zum Effekt der körperlichen Bewegungstherapie vor. Demgegenüber wurde sowohl bei Gesunden, als auch bei einer Vielzahl von Erkrankungen, insbesondere bei Patienten mit chronischer Linksherzinsuffizienz (Hambrecht 2000, 2002) und der koronaren Herzerkrankung (Schuler 2002) in randomisierten Studien der positive Einfluss regelmäßiger körperlicher Aktivität auf die Prognose, die Leistungsfähigkeit, das subjektive Wohlbefinden, die oxidative Kapazität der Skelettmuskulatur, die Endothelfunktion sowie die Ventilation nachgewiesen. Dabei hat die Bewegungstherapie bei Linksherzinsuffizienz eine geringe therapeutische Breite. Bei zu geringen Belastungen werden nicht die erwünschten Wirkungen erzielt, bei zu hohen Belastungen sind die Patienten einem erhöhten Risiko ausgesetzt (Hambrecht 2002). Zur Einschätzung der Belastungsintensität wird daher die Festlegung des "Trainingspulses" bei 60% der VO2max. empfohlen (Hambrecht 2002). Als Ausschlusskriterien für körperliches Training gelten folgende Erkrankungen: aktive Myokarditis, instabile Angina Pectoris, belastungsinduzierte ventrikuläre Rhythmusstörungen, dekompensierte Herzinsuffizienz, bedeutsame Herzvitien und die hypertrophe obstruktive Kardiomyopathie.

Auch die Atemtherapie hat sich in den letzten Jahren in der Rehabilitation von Patienten mit Lungenerkrankungen etabliert (Goldstein 1994, Ries 1997,Güell 2000). Die verschiedenen Techniken sollen unter anderem die Ventilation verbessern, die Atemmuskulatur kräftigen, den Thorax mobilisieren oder sekretolytisch wirken (Göhring 2001, Ehrenberg 1998, Beh 1999). Beim chronischen Lungenhochdruck liegen bislang keine Untersuchungen zur Atemtherapie vor.

Das Mentale Gehtraining wurde aus dem Hochleistungssport transferiert und im Hinblick auf die Anwendung in der Rehabilitation modifiziert. Im Sport ist die lern- und leistungssteigernde Wirkung des Mentalen Trainings eindeutig belegt (vgl. u.a. Feltz & Landers 1983, Driskel et al 1994) und das Verfahren theoretisch fundiert (Heuer 1985). Auch in sportexternen Anwendungsbereichen, wie bspw. der invasiven Medizin, wurde die positive Wirkung des Mentalen Trainings bereits nachgewiesen (siehe ausführlich 1.2.). Diese positiven Resultate bestätigen sich auch für das Mentale Gehtraining. So konnte die positive Therapiewirkung des Mentalen Gehtrainings im Hinblick auf die Optimierung der Gehbewegung für Patienten nach Hüft-Totalendoprothese eindeutig wissenschaftlich nachgewiesen werden. Zudem wurde dieses Therapieverfahren bei weiteren orthopädischen Erkrankungen in der Praxis erfolgreich angewendet (siehe ausführlich 1.2.). Ob das Mentale Gehtraining auch zur Optimierung der Gehbewegung bei Patienten mit chronischem Lungenhochdruck beitragen kann, wurde bisher noch nicht untersucht.

**Literatur:**

Beh D., Atemgymnastik, München: BLV; 1999

Driskell, J., Copper, C. & Moran, A. Does mental practice enhance performance? Journal of Applied Psychology 1994; 79, 481-492.

Ehrenberg H., Atemtherapie in der Physiotherapie / Krankengymnastik, München: Pflaum; 1998

Feltz, D.L. & Landers, D.M. The effects of mental practice on motor skill learning and perfor­mance: A meta-analysis. Journal of Sport Psychology 1983; 5, 25-57.

Goldstein RS, Gort EH, Stubbing D., Avendano MA, Guyatt GH Randomised controlled trial of

respiratory rehabilitation. Lancet. 1994; 344: 1394-1397

Göhring H., Atemtherapie – Therapie mit dem Atem, Stuttgart: Thieme 2001

Güell R, Casan P, Belda J, Sangenia M, Morante F, Guyatt g, Sanchis J.Long-term Effects of outpatient rehabilitation of COPD; Chest. 2000;117:976-983

Hambrecht R, Schuler G. Die körperliche Belastbarkeit des herzinsuffizienten Patienten - Training bei Herzinsuffizienz? Internist 2000; 41:269-275

Hambrecht R. Belastung und Belastbarkeit bei chronischer Herzinsuffizienz. Herz 2002; 27:179-186

Heuer, H. Wie wirkt mentale Übung? Psychologische Rundschau 1985; 36, 191-200.

Rich S, Dantzker DR, Ayres SM, et al. Primary pulmonary hypertension. A national prospective study. Ann Intern Med. 1987;107:216-223

Ries AL. Pulmonary rehabilitation:evidence-based guidelines. Chest.1997; 112: 1363-1396

Runo J R, Loyd JE. Primary pulmonary hypertension. Lancet 2003;361:1533-1544

Schuler G. Primäre und sekundäre Prevention: körperliche Aktivität. Z Kardiol 2002;91:30-39

Seeger W, Schäfers HJ. Pulmonale Hypertonie. Blackwell Wissenschaftsverlag, Berlin 2001

## 1.2. Eigene Vorarbeiten

Seit 1996 wurde in Zusammenarbeit zwischen der Abteilung für Allgemeine Klinische und Psychosomatische Medizin und der Abteilung für Kardiologie, Angiologie und Pneumologie bei herzinsuffizienten Patienten neben einer umfassenden somatischen Abklärung eine breite psychische Basisdiagnostik zur Lebensqualität und emotionaler Belastung durchgeführt. Daraus entstand eine fundierte Wissensbasis in Auswahl und Anwendung von Instrumenten der psychischen Basisdiagnostik. Aus dem unter Leitung von Prof. Haass etablierten Herzinsuffizienzregister, welches mittlerweile mehr als 1.000 kontinuierlich verfolgte und gut charakterisierte herzinsuffiziente Patienten enthält, konnten gemeinsam zahlreiche neue Erkenntnisse zu Krankheitsverlauf bestimmenden Parametern gewonnen werden. Bislang vorliegende komplizierte Risikostratifizierungsverfahren konnten durch praxistaugliche, für das Monitoring besser geeignete Konzepte ersetzt werden. Seit 1996 werden Patienten mit schwerer therapierefraktärer Herzinsuffizienz, die auf der Warteliste für eine Herztransplantation stehen, interdisziplinär (Kardiologie, Psychosomatik, Sozialarbeit und Herzchirurgie) betreut und erhalten in einem Stufenprogramm angepasste Therapieangebote. Aus diesem Ansatz entstand eine der bundesweit größten Selbsthilfegruppen für Herztransplantierte (Herztransplantation Südwest e. V.).

In einem interdisziplinären stationär-ambulanten Interventionsprogramm für schwer herzinsuffiziente Patienten unter der Beteiligung der beiden genannten Abteilungen und der Rehabilitationsklinik Heidelberg-Königstuhl konnte neben einer Verbesserung der kardio-pulmonalen Leistungsfähigkeit die nachhaltige Verbesserung der Compliance, Selbstwirksamkeit und der LQ der Patienten erreicht werden. Das Programm wurde von der Ethikkommission genehmigt und im Mai 2001 mit dem Sonderpreis für „Patient education“ der Bayerischen Landesbank ausgezeichnet.

Die Gruppe von Dr. Ekkehard Grünig arbeitet seit 1997 mit Patienten mit Lungenhochdruck und hat zeigen können, daß Höhenlungenödemanfällige (Grünig 2000) als auch Familienmitglieder, die eine genetische Disposition zum primärem Lungenhochdruck aufweisen (Grünig 2002) mittels Stress-Dopplerechokardiographie identifiziert werden. Zudem wurde ein zweiter Genort für den Lungenhochdruck auf dem Chromosom 2q31 lokalisiert (Janssen 2002, Rindermann 2003). Im Rahmen dieser Projekte wurde ein nationales (Zusammenarbeit mit Giessen, Hannover, Berlin, Leipzig, Dresden, Homburg, Freiburg, München) und internationales (Nashville, Cincinnati, USA; Leicester, England, Polen, Frankreich, Italien, Belgien) Netzwerk zur Genetik und Frühdiagnostik der pulmonalen Hypertonie aufgebaut und der von Heidelberg initiierte EU-Antrag „Early diagnosis and analysis of the genetic causes of primary pulmonary hypertension (PPH), a rare and life-threatening disease“ von der EU-Kommission bewilligt. Die geplanten klinischen Untersuchungsmethoden sind daher in der Kardiologie gut etabliert. Grünig ist Mitbegründer der gemeinsamen Arbeitsgruppe für PH und Mitautor der Leitlinien für PH der deutschen Gesellschaft für Kardiologie, Kinderkardiologie und Pneumologie. Zudem wurde er für Juni 2003 in die Task Force des Weltkongresses für Lungenhochdruck berufen.

Neben dem Einsatz in der Rehabilitation (vgl. dazu auch weitere Veröffentlichungen der Heidelberger Arbeitsgruppe zur Rehabilitation nach Sportverletzungen, bspw. Hermann & Eberspächer 1994) wird das Mentale Training auch in anderen Anwendungsgebieten erfolgreich durchgeführt. So hat dieses Trainingsverfahren seinen Ursprung im Hochleistungssport, wo es in fast allen Sportarten zur Optimierung von Bewegungen eingesetzt wird (vgl. Eberspächer 2001, Immenroth et al. in Vorbereitung). Neuerdings wird das Mentale Training von der Heidelberger Arbeitsgruppe auch in sportexterne Arbeits-, Betriebs- und Organisationsprozesse, bspw. die invasive Medizin und die Luftfahrt, transferiert und dort wissenschaftlich evaluiert. Auch hier zeigten sich eindeutig positive Wirkungen dieses Trainingsverfahrens (Eberspächer & Immenroth 1999, Immenroth 2003, Immenroth et al. in Vorbereitung).

**Publikationen:**

Eberspächer, H. Mentales Training. München: sport­inform 2001.

Eberspächer, H. & Immenroth, M. Mentales Training - hilft es auch dem modernen Chirurgen? Zentralblatt für Chirurgie 1999; 124, 895-901.

Hermann, H.-D. & Eberspächer, H. Psychologisches Auf­bautraining nach Sportver­letzungen. Mün­chen: BLV 1994.

Immenroth, M. Mentales Training in der invasiven Medizin. Hamburg: Kovac 2003.

Immenroth, M., Eberspächer, H. & Hermann, H.-D. Training kognitiver Fertigkeiten. In J. Beckmann & M. Kellmann (Hrsg.), Enzyklopädie der Psychologie. D/V/2, Anwendungen der Sportpsychologie. Göttingen u.a.: Hogrefe (in Vorbereitung).

Juenger J, D. Schellberg, S. Kraemer, A. Haunstetter, C. Zugck, W. Herzog and M. Haass. Health related quality of life in patients with congestive heart failure: comparison with other chronic diseases and relation to functional variables. Heart 2002;87, 235-241.

Juenger J, D. Schellberg, T. Mueller-Tasch, G. Raupp, C. Zugck, A. Haunstetter, S. Zipfel, W. Herzog and M. Haass. Depression increasingly predicts mortality in the course of congestive heart failure. Eur J Heart Fail, submitted (2003).

Mayer, J. Mentales Training - ein salutogenes Therpieverfahren zur Bewegungsoptimierung. Hamburg: Kovac 2001.

Mayer, J., Görlich, P. & Eberspächer, H. Mentales Gehtraining. Heidelberg: Springer 2003.

Meyer FS, M. M. Borst, C. Zugck, A. Kirschke, D. Schellberg, W. Kuebler and M. Haass. Respiratory muscle dysfunction in congestive heart failure: clinical correlation and prognostic significance. Circulation 2001;103, 2153-2158.

Zipfel S, Schneider A, Wild B, Loewe B, J. Juenger, M. Haass, Sack F-U, G. Bergmann and W. Herzog. Effect of depressive symptoms on survival after heart transplantation. Psychosom Med 2002;64:740-747.

Zugck C, C. Kruger, R. Kell, S. Korber, D. Schellberg, W. Kubler and M. Haass Risk stratification in middle-aged patients with congestive heart failure: prospective comparison of the Heart Failure Survival Score (HFSS) and a simplified two-variable model. Eur J Heart Fail 2001;3,577-585.

Grünig E, Mereles D, Hildebrandt W, et al. Stress-Doppler-echocardiography for identification of susceptibility to high altitude pulmonary edema. J Am Coll Cardiol. 2000;35:980-987.

Grünig E, Janssen B, Mereles D, et al. Abnormal pulmonary artery pressure response in asymptomatic carriers of primary pulmonary hypertension gene. Circulation 2002; 102: 1145-1150

Janssen B, Rindermann M, Barth U, Miltenberger-Miltenyi G, Mereles D, Abushi A, Seeger W, Kübler W, Bartram CR, Grünig E. Linkage analysis in a large family with Primary Pulmonary Hypertension (PPH): genetic heterogeneity and a second PPH locus on 2q31-32. Chest 2002, 121:54-56

Rindermann M, Grünig E, Hippel A, et al. Primary Pulmonary Hypertension may be a heterogeneous disease with a Second Locus on Chromosome 2q31. JACC 2003, 41:2237-2244.

**2. Ziele der Studie/Zielkriterien**

## 2.1. Primäre Zielkriterien sind:

## 2.1.1. Veränderung der 6-Minuten-Gehstrecke als Differenz zwischen dem Wert der Eingangsuntersuchung und dem Wert nach 3 Wochen (am Ende der Reha)

## 2.1.2. Veränderung der Lebensqualität nach dem SF-36: Differenz zwischen Eingangsuntersuchung und einem Monat nach Reha.

## 2.2. Sekundäre Zielkriterien sind

## 2.2.1. Veränderung der 6-Minuten-Gehstrecke: Differenz zwischen dem Wert der Eingangsuntersuchung und 3 Monaten nach Reha.

## 2.2.2. Veränderung der Lebensqualität nach dem SF-36: Differenz zwischen Eingangsuntersuchung und 3 Monaten nach Ende der Reha.

## 2.2.3. Körperliche Belastbarkeit im Liegendergometer (Watt) = a) Differenz zwischen dem Wert der Eingangsuntersuchung und dem Wert nach 3 Wochen (Ende der Reha) sowie

## b) dem Wert nach 3 Monaten.

## 2.2.4. Hämodynamische Parameter: Größe und Pumpfunktion des rechten Ventrikels,

## Verbesserung des systolischen PA-Druckes in Ruhe und unter Belastung= a) Differenz vom Wert der Eingangsuntersuchung und dem Wert nach 3 Wochen am Ende der Reha und

## b) dem Wert nach 3 Monaten.

2.2.5. Änderung der Perfusionsparameter im MRT

2.2.6. WOMEC

2.2.7. Änderung des BNP-Wertes Ausgangsuntersuchung, Ende der Reha und -3 Monate nach Reha.

**2.3 Weitere Ziele sind**

## 2.3.1. Häufigkeit und Dauer stationären Aufenthalte, Vermindertes Auftreten bedrohlicher Ereignisse

2.3.2. Verbesserung der Ein-Jahres Überlebensrate.

2.3.3. Die für die Anwendung beim Lungenhochdruck nicht spezifizierte „Atem- und

Bewegungstherapie“ soll im Rahmen dieser Studie weiterentwickelt und auf die

Bedürfnisse dieser Patientengruppe hin ausgerichtet werden.

2.3.4. Bei diesem Projekt soll überprüft werden, inwieweit das "mentale Gehtraining" in die

Rehabilitationsmaßnahmen einbezogen werden kann und für die Patienten verständlich und umsetzbar ist. Die Akzeptanz des Trainings soll mittels Fragebogen des Instituts für Sportwissenschaft überprüft werden.

2.3.5. Die Atem- und Bewegungstherapie sowie das mentale Gehtraining bei Lungenhochdruck soll zunächst bei 30 Patienten durchgeführt werden. Anschließend ist es geplant, das Projekt in Zusammenarbeit mit weiteren klinischen Zentren, insbesondere der Pneumologie der Thoraxklinik Rohrbach (Prof. Herth), der Universtität Giessen (Prof. Seeger, PD Dr. Horst Olschewski, Frau Dr. Ghofrani), der Universität Hannover (Prof. Marius Höper) sowie der Universität Dresden (Dr. Michael Halank) bei mindestens 100 Patienten durchzuführen.

2.3.6. Es soll ein PH-spezifisches Rehabilitationsprogramm etabliert werden, das auch in andere Rehabilitationskliniken durchgeführt werden kann.

2.3.7. Veränderung der Depressivität und Angst: Patients Health Questionaire

## 2.3.8. Verbessertes Befinden: (NYHA-Klasse, Borg-Skale)

2.3.9. 6-Minuten Gehstrecke bei der Trainingsgruppe jede Woche

# 3. Zu prüfendes Verfahren (nur soweit einschlägig)

- keine, alle eingesetzten Trainingsverfahren sind etablierte Maßnahmen, wie die Atemtherapie oder das Intervalltraining

# 4. Studiendesign(-typ)

Es handelt sich um eine prospektive, multizentrische, kontrollierte, randomisierte Studie, bei der die Patienten mit invasiv gesicherter PH in 2 Gruppen randomisiert werden. Die Patienten der Gruppe A erhalten neben ihrer medikamentösen Therapie eine konventionelle Kur, bei der - wie bislang bei PH üblich- körperliche Belastung vermieden werden soll. Patienten in Gruppe B wird neben der konventionellen Kur zusätzlich eine Atem- und Bewegungstherapie sowie ein mentales Gehtraining angeboten.

# 5. Randomisierungsverfahren/ggf. -plan

**Beschreibung/Begründung der Randomisierung**

Die Zuordnung der Patienten/Probanden zu der Atem- und Bewegungstherapie und mentalem Training erfolgt nach dem Zufallsprinzip. Dies hat den Zweck, eine möglichst hohe wissenschaftliche Aussagekraft der Untersuchung zu erreichen. Die Randomisierung erfolgt nach der Zusage der Kostenübernahme durch den zuständigen Träger nach Zufallsprinzip und nach dem Blockrandomisierungsverfahren. Es werden folgende Gruppen gebildet:

**A) Konventionelle Therapiegruppe:** Diese Gruppe enthält Patienten, die eine konventionelle Kur erhalten. Die Kurmaßnahmen umfassen gesunde Ernährung, Massagen, Entspannungsbäder, leichte Spaziergänge, Unterhaltungselemente und sollen einen Urlaubscharakter haben und der Erholung dienen. Diese Patienten erhalten keine Elemente der spezifischen Atem- und Bewegungstherapie und des mentalen Trainings. Zudem erhalten alle Gruppen Beschäftigungstherapie. Dabei sollen die Therapieelemente stundenweise entsprechend zwischen den Gruppen abgeglichen werden. Es ist darauf zu achten, dass die Gesamttherapiezeit bestehend aus den spezifischen Therapiemaßnahmen und der Beschäftigungstherapie (wie Vorträge, Basteln, Spiele) bei allen Gruppe gleich ist.

**B) Interventionsgruppe:** Konventionelle Therapie plus spezifische Atem- und Bewegungstherapie plus mentales Gehtraining.

Die Patienten werden so zur stationären Rehabilitation einbestellt, daß Patienten verschiedener Gruppen nicht gleichzeitig behandelt werden. Damit soll erreicht werden, dass sowohl die betreuenden Therapeuten und "Trainer" als auch die Patienten verschiedene Therapieangeboten durchführen können, ohne durcheinander zu kommen. Zudem wurde dieses Verfahren gewählt, um eine gegenseitige Beeinflussung der Patienten und einen Kohorteneffekt auszuschließen. Die Patienten werden über die Zuordnung zu den Gruppen informiert. Den Patienten der konventionellen Gruppe wird ein erneuter Aufenthalt in der Rehabilitationsklinik 3 Monate später angeboten, bei der die gleichen Maßnahmen wie bei der Gruppe B durchgeführt werden.

# 6. Einschlusskriterien

Eingeschlossen werden Patienten mit invasiv gesicherter chronischer PH, die von spezialisierten Ärztinnen/Ärzte eine vollständige diagnostische Abklärung gemäß der WHO-Klassifikation an einem Zentrum für Lungenhochdruck erhalten haben und seit 3 Monaten unter intensivierter medikamentöser Therapie eingestellt und stabil sind. Zudem müssen die Patienten eine von den Kranken- bzw. Rentenkassen genehmigte Kur in der Rehabilitationsklinik Königstuhl durchführen. Die Patienten sollten mindestens 18 und höchstens 75 Jahren alt sein und in NYHA-Klasse II-III sein. Im Rahmen dieser Studie sollen insgesamt ca. 30 Patienten einbezogen werden. Fünfzehn Patienten in Gruppe A, konventionelle Kur, weitere fünfzehn Patienten der Gruppe B, konventionelle Kur + Atem- Bewegungstherapie und mentales Gehtraining.

# 7. Ausschlusskriterien

Patienten mit Zeichen der Rechtsherzdekompensation, Gangstörung, NYHA-Klasse IV, unklare Diagnosen, keine vorausgegangene invasive Abklärung, akute Erkrankungen, Infektionen, Fieber.

Weitere Ausschlusskriterien sind folgende Erkrankungen: aktive Myokarditis, instabile Angina Pectoris, belastungsinduzierte ventrikuläre Rhythmusstörungen, dekompensierte Herzinsuffizienz, bedeutsame Herzvitien und hypertrophe obstruktive Kardiomyopathie, Schwangerschaft.

# 8. Studienablauf

Die geplante Atem- und Bewegungstherapie wird im Rahmen eines regulären, von den Kassen finanzierten 3-wöchigen Kuraufenthaltes in der Rehaklinik Königstuhl, Heidelberg begonnen. Nach Zusage der Kostenträger werden die Patienten in 2 Gruppen randomisiert. Patienten der Gruppe A erhalten eine konventionelle Kur, bei körperliche Belastung vermieden wird. Im Vordergrund der Kur stehen hier gesunde Ernährung, Massagen, Entspannungsbäder, leichte Spaziergänge, Urlaubscharakter und Erholung. Diese Patienten erhalten keine Elemente der spezifischen Atem- und Bewegungstherapie und des mentalen Trainings. In der Gruppe B erhalten die Patienten neben der konventionellen Therapie spezifische Atem- und Bewegungstherapie plus mentales Gehtraining. Am Ende der Kur soll gemeinsam mit dem Patienten ein individuelles Programm für die Durchführung zu Hause bzw. bei der ambulanten Physiotherapie erarbeitet werden. Die Patienten werden über die Zuordnung zu den Gruppen informiert. Den Patienten der konventionellen Gruppe wird ein erneuter Aufenthalt in der Rehabilitationsklinik 3 Monate später angeboten, bei der die gleichen Maßnahmen wie bei der Gruppe B durchgeführt werden.

Zu Beginn und am Ende der Rehabilitationsmaßnahme, sowie nach 3 Monaten soll eine klinische Untersuchung vorgenommen werden, bei der die Zielparameter erfasst werden. Diese Untersuchungen finden im Rahmen regulärer ambulanter Kontrolltermine bei den beteiligten Zentren statt. Zu Beginn der Rehabilitationsmaßnahme und nach 3 Monaten wird eine MRT Untersuchung durchgeführt, die in dem Antrag 110/2003 „Funktionelle und morphologische Evaluierung von Patienten mit Pulmonaler Hypertonie (PH) mittels Magnetresonanztomographie (MRT)“ beschrieben und von der Ethikkommission bewilligt wurde. Bei der Eingangsuntersuchung soll der "Trainingspuls" anhand der VO2max in der Spiroergometrie bestimmt werden. Zudem soll durch die Bestimmung des systolischen PA-Druckes die individuelle rechtsventrikuläre Belastung bei der Bewegungstherapie abgeschätzt werden. Anhand der Selbsteinschätzung der Patienten und der in der Eingangsuntersuchung erhobenen Daten wird das Programm der Atem- und Bewegungstherapie für jeden einzelnen Patienten der Gruppe B individuell festgelegt und während des Kuraufenthaltes weiter überprüft und ggf. modifiziert. Dabei soll größte Aufmerksamkeit darauf gelegt werden, den Patienten keine größeren Belastung zuzumuten, als solche, die sie tolerieren können und ohnehin im Alltag vornehmen. Hierzu muß das Befinden des Patienten täglich engmaschig protokolliert und erfasst werden, um Anzeichen einer Überforderung rechtzeitig zu erkennen. Bei der Bewegungstherapie soll der "Trainingspuls" nicht die Herzfrequenz übersteigen, die bei 60% der maximalen symptom-limitierten Belastung erzielten Sauerstoffaufnahme entsteht.

Während der nachstationären Phase wird jeder Patient der Interventionsgruppe B alle 2-4 Wochen angerufen, um das Befinden bzw. die Einhaltung der spezifischen Maßnahmen abzufragen. Dabei sollen auch die individuellen Programme immer wieder angeregt werden. Für die Kontrolluntersuchung nach 3 Monaten wird zumindest den weiter anreisenden Patienten zwei kostenlose Übernachtungen sowie die Übernahme der Fahrtkosten angeboten. Bei der Abschlusskontrolle reisen die Patienten Sonntags abends an, Montags morgens wird die 6-Minuten-Gehstrecke gemessen, danach erfolgt die Untersuchungen mittels Stressechokardiographie. Nachmittags wird das Übungsprogramm und Aufbautraining nochmals besprochen. Am zweiten Tag erfolgt morgens die MRT-Untersuchung, danach die Heimreise.

**9. Therapieelemente im Rahmen der stationären Rehabilitation**

**9.1. Konventionelle Therapie:** zumindest eine tägliche Arztvisite, Massagen, Entspannungsübung, Bäder, leichte kurze Spaziergänge. Zudem erhält diese Therapie die folgenden Elemente:

**9.1.1. Psychologische Gespräche:** werden den Patienten 2 mal pro Woche angeboten (ggf. gemeinsam mit dem Partner/der Partnerin). Thema: Bedeutung der Erkrankung in Bezug auf Familie, Partnerschaft und Beruf. Dabei sollen positiver Ansätze zur Bewältigung der Erkrankung erarbeitet werden. Zudem werden Entspannungstherapien angeboten.

**9.1.2. Progressive Muskelentspannung:** Die Patienten können zweimal wöchentlich an Gruppenterminen zum Erlernen und Einüben der progressiven Muskelentspannung teilnehmen.

**9.1.3. Weitere Maßnahmen/Beschäftigungsangebote:** Als unterstützende physikalisch-balneologische Maßnahmen erhalten die Pat. bei Bedarf noch leichte Rückenmassagen und Wärmeanwendungen sowie ggf. auch Inhalationstherapie. Zudem bestehen Angebote wie Malen, Plastizieren, Basteln, Spiele-Abende, die dem allgemeinen Wohlbefinden dienen.

**9.2. Spezifische Atem- und Bewegungstherapie:** Diese Therapie wird durch sämtliche unter 9.1. beschriebenen konventionellen Maßnahmen ergänzt.

**9.2.1. Spezifische Atemtherapie:** siehe auch Beiblatt, in dem die Übungen beschrieben sind. Bei der Atemtherapie werden standardisierte Techniken verwendet, mit denen eine bessere Ventilation erreicht werden soll. Hierzu sollen die Patienten häufiger Zwerchfell-atmung durchführen und atemerleichternde Haltungen einnehmen. Zudem soll durch die Therapie die Atemmuskulatur gekräftigt und die Thoraxbeweglichkeit verbessert werden. Dabei werden auch kleine technische Hilfsmittel, wie der „Atemtrainer“ eingesetzt, mit dem die Patienten die Inspiration gegen eine geringen, individuell zu dosierenden Atemwiderstand üben. Die Patienten sollen während der Kur die für sie hilfreichen Atemtechniken erlernen und danach im häuslichen Rahmen fortführen.

**9.2.2. Ergometerintervalltraining:** Mit speziellen Fahrradergometern wird im Wechsel 1 Minute „aktive Erholung“ mit 0 – 25 Watt und jeweils für 30 Sekunden eine höhere Wattstufe (z.B. 25-50 Watt) trainiert, um für die Beinmuskulatur Trainingsreize zu setzen und dennoch das Herz möglichst wenig zu belasten. Die Intensität des Ergometerintervalltrainings wird nach der Herzfrequenz gesteuert, wobei als Trainings-Herzfrequenz 60 % der im Rahmen der Spiroergometrie erreichten maximalen Herzfrequenz angesetzt wird.

**9.2.3. Begleitete Spaziergänge:** Die Patienten unternehmen ebenerdige Spaziergänge in Begleitung einer Bewegungstherapeutin. Die Geschwindigkeit wird dabei nach Herzfrequenz angepasst (60 % der max. Herzfrequenz bei Vo2max.). Die Herzfrequenz wird mittels Polar-System überwacht.

**9.2.4. Leichtes Muskelaufbautraining einzelner Muskelgruppen:** Es werden mit sehr geringen Gewichten (z.B. 500 g) einzelne Muskelgruppen (z.B. ein Oberarm) trainiert. Danach erfolgt der Wechsel auf die andere Seite bzw. auf weitere Muskelgruppen. Dabei wird besonderer Wert auf die richtige Atemtechnik (keine Pressatmung) gelegt.

**9.3. Mentales Gehtraining:** Diese Therapie wird durch sämtliche unter 9.1. und 9.2. beschriebenen konventionellen Maßnahmen ergänzt. Bei diesem Verfahren sollen die Patienten die Bewegungsabläufe gedanklich durchführen. Im Rahmen dieses mentalen Gehtrainings sollen die Bewegungsabläufe weiter analysiert und optimiert werden.

**10. Kontrollparameter (siehe Begleitbogen CRF)**

Sämtliche Kontrollparameter sollen bei der Eingangsuntersuchung, am Ende der Reha und nach 3 Monaten erhoben werden. Nach einem Jahr soll der Fragebogen SF-36 verschickt und Fragen zur Befindlichkeit telefonisch erhoben werden.

**10.1. Anamnese:** Bei der Anamnese wird der Patient u.a. nach seinem körperlichen Leistungsvermögen und Befinden im Alltag befragt.

**10.2. Protokoll der Befindlichkeit und der körperlichen Belastbarkeit:**

- Fragebogen zur Lebensqualität: SF-36
- NYHA - Klassifikation
- Borg-Skale

**10.3. Prüfung der körperlichen Belastbarkeit**

- 6-Minuten Gehtest, sollte am zweiten und am vorletzten Tag der Kur, sowie nach 3 Monaten durchgeführt werden. Der Gehtest sollte jeweils morgens immer in individuell gleichem Abstand nach Medikamenteneinnahme und Frühstück stattfinden. Vor und nach dem Gehtest wird die Herzfrequenz sowie der systemische Blutdruck gemessen und dokumentiert.
- VO_2_max. in der Spiroergometrie
- Belastungsstufe im Liegendergometer
- Maximale Herzfrequenz mal maximaler systolischer Blutdruck während Belastung

**10.4. Hämodynamische Parameter**

**Stress-Doppler-Echokardiographie:** Diese Untersuchung wird in der Kardiologie durch Dr. Grünig, Dr. Mereles und Frau Sandra Kreuscher durchgeführt. Zunächst wird ein Ruhe EKG und ein Echokardiogramm in Ruhe vorgenommen. Dann beginnt die Belastung mit 25W und steigert sich alle 2 Minuten um 25W. Während der Belastung wird alle 2 Minuten ein EKG geschrieben und der Blutdruck gemessen. Zudem wird unter der Belastung eine Echokardiographie durchgeführt, bei der neben der Herzkraft auch der Lungengefäßdruck bestimmt wird.

**Spiroergometrie:** Während der Stressechokardiographie wird gleichzeitig eine Spiroergometrie durchgeführt. Dazu atmet der Proband durch ein Mundstück und die Nase wird durch eine Nasenklammer verschlossen.

MRT: Diese Untersuchung wird von Dr. Ley im DKFZ gemäß dem im Antrag 110/2003 genannten Angaben bei der Eingangsuntersuchung, nach 3 Wochen und nach 3 Monaten durchgeführt.

10.5. Laborparameter: Im Rahmen des 3-wöchigen Kuraufenthaltes, werden die üblichen Routinelaborparameter wie Hb, Ery, HK, Leuko, Thrombo, Reti, BKS, Ges.-Eiw., TSH, Chol., LDL-Chol., HDL-Chol., Triglyzeride, Harnsäure, Harnstoff, Krea, Na, K, Cl, Ca, Mg, Eisen, Amylase, Lipase, Bili, SGOT, SGPT, yGT, LDH, CK, alk. Phosphatase, NBZ, TnT und BNP abgenommen. BNP und TnT sollte bei der Eingangsuntersuchung, nach 3 Wochen und 3 Monaten bestimmt werden.

# 11. Begleittherapie

Eine medikamentöse Begleittherapie ist erlaubt. Die Patienten nehmen ihre Medikamente wie bisher ein. Die medikamentöse Therapie sollte dabei nicht verändert werden, soweit nicht aus klinischen Gründen erforderlich.

# 12. Abbruchkriterien

1. Befinden des Patienten verschlechtert sich
2. Zeichen der Rechtsherzdekompensation
3. Synkope
4. Symptomatische ventrikuläre Tachykardie
5. Verschlechterung der NYHA-Klasse
6. Eintreten einer der oben genannten Ausschlusskriterien.

Die gesamte Studie wird abgebrochen, wenn bei mehr als 3 Studienteilnehmern der Gruppe B Abbruchkriterien eingetreten sind.

# 13. Ethische und rechtliche Aspekte

## 13.1. Ethische Grundlagen

Die Untersuchung wird in Übereinstimmung mit der Deklaration von Helsinki in der aktuellen Fassung von 1996 durchgeführt. Die Teilnahme der Patienten/Probanden an der Untersuchung ist freiwillig; die Zustimmung kann jederzeit, ohne Angabe von Gründen und ohne Nachteile für die weitere medizinische Versorgung, zurückgezogen werden.

### 13.2. Patienten-/Probandeninformation/Einverständniserklärung

Die Patienten/Probanden werden vor Studienbeginn schriftlich und mündlich über Wesen und Tragweite der geplanten Untersuchungen und Maßnahmen, insbesondere über den möglichen Nutzen für Ihre Gesundheit und eventuelle Risiken, aufgeklärt. Ihre Zustimmung wird durch Unterschrift auf der Einwilligungserklärung dokumentiert. Für die Untersuchung mit MRT erhalten die Patienten nochmals einen gesonderten Aufklärungsbogen und Einverständniserklärung, die Bestandteil des Antrages 110/2003 ist.

Bei Rücktritt von der Studie wird bereits gewonnenes (Daten-) Material vernichtet oder beim Proband/Patient angefragt, ob er mit der Auswertung des Materials einverstanden ist.

## 14. Rechtliche Grundlagen

### 14.1. Votum der Ethikkommission

Der Untersuchungsplan wird vor Studienbeginn der Ethikkommission der Medizinischen Fakultät Heidelberg zur Begutachtung vorgelegt. Es wird nicht mit dem Einschluss von Probanden/Patienten begonnen, bevor nicht das schriftliche Votum der Ethikkommission vorliegt.

### 14.2 Datenschutz/Einblick in Originalkrankenunterlagen

Die Namen der Patienten und alle anderen vertraulichen Informationen unterliegen der ärztlichen Schweigepflicht und den Bestimmungen des Bundesdatenschutzgesetzes (BDSG). Eine Weitergabe von Patientendaten erfolgt ggf. nur in anonymisierter Form.

**14.3. Patientenversicherung**

Die Atem- und Bewegungstherapie wird initial während eines 3 wöchigen Kuraufenthaltes in der Rehabilitationsklinik Königstuhl, Heidelberg der Patienten vorgenommen, der von den Kranken- bzw. den Rentenkassen bezahlt wird und in dessen Rahmen die Patienten über das Krankenhaus versichert werden. Anschließend sollen die Patienten diese Übungen, soweit sie Ihnen gut tun zu Hause weiter fortführen. Die geplanten Untersuchungen werden im Rahmen eines regulären ambulanten Kontrolltermins in Heidelberg durchgeführt.

# 15. Unterschriften

# Dr. med. Ekkehard Grünig Sandra Kreuscher, Physiotherapeutin

# Appendices

**1. Einverständniserklärung**

**2. Patienteninformation**

**3. Atemtherapie, Beschreibung der Maßnahmen**

**4. CRF**

**5. SF-36**

**6. 6-Minuten-Gehtest**

**7. Therapiepläne**

**Einverständniserklärung**

Atem- und Bewegungstherapie als unterstützende Maßnahme bei Patienten mit pulmonal arterieller Hypertonie

Ich bin über Sinn, Bedeutung und Verlauf der Studie sowie über mögliche Belastungen und Risiken anhand der schriftlichen Patienten-/Probanden-Information, die ich erhalten habe, und durch Herrn / Frau ________________________ aufgeklärt worden. In diesem Zusammenhang sind mir alle meine Fragen vollständig beantwortet worden

Ich stimme der Teilnahme an der Studie freiwillig zu. Ich weiß, dass ich diese Zustimmung ohne Angabe von Gründen jeder­zeit und ohne Nachteile für meine weitere medizinische Versorgung widerrufen kann.

Bei Rücktritt von der Studie bin ich mit der Auswertung meines (Daten-) Materials

einverstanden ⭘ja ⭘nein

**Ich wurde darüber aufgeklärt und stimme zu, dass die im Rahmen dieser Studie erhobenen Daten in anonymisierter Form dokumentiert und ggf. weitergegeben werden.**

**Dritte erhalten keinen Einblick in Originalkrankenunterlagen.**

**Name des Patienten:**

**Geburtsdatum:**

**Datum Unterschrift des Patienten Unterschrift des Prüfarztes**

**Patienteninformation: Atem- und Bewegungstherapie bei chronischem Lungenhochdruck**

Liebe Patienten!

Die schwere pulmonale Hypertonie (PH) ist eine Erkrankung, die oft erst bemerkt wird, wenn der Lungenhochdruck schon weit fortgeschritten ist. In den letzten Jahren hat sich die medikamentöse Therapie stetig weiterentwickelt. Als begleitende Maßnahmen wird häufig körperliche Schonung empfohlen. Einige Patienten haben demgegenüber davon berichtet, dass Ihnen regelmäßige körperliche Betätigung gut tut. Zudem gibt es Untersuchungen bei anderen Erkrankungen, wie der Linksherzschwäche, die zeigen, dass vorsichtiges, regelmäßiges körperliches "Training" die körperliche Leistungsfähigkeit, die Lebensqualität und die Prognose der Erkrankung verbessern kann. Die Atemtherapie ist ebenfalls bislang bei vielen chronischen Erkrankungen der Lunge, wie dem Asthma Bronchiale, eine gut etablierte Maßnahme, die die Atemmuskulatur stärken und die Belüftung der Lunge verbessern soll. Weder die Atem- noch die Bewegungstherapie wurden bislang bei Patienten mit Lungenhochdruck systematisch eingesetzt und in ihrer Wirkung untersucht.

**Ziel dieses Rehabilitationsprojekt** ist, zu untersuchen, ob und inwieweit eine vorsichtige Atem- und Bewegungstherapie die medikamentöse Therapie ergänzen kann und das Befinden, die Lungengefäßdrucke, die Größe des rechten Herzens und die 6-Minuten Gehstrecke bei Patienten mit Lungenhochdruck verändert.

**Studienablauf:** Bei diesem Rehabilitationsprojekt werden 2 Gruppen gebildet. In Gruppe A sind die Patienten, die zunächst eine konventionelle Kur erhalten (siehe unten). Patienten in Gruppe B erhalten zusätzlich eine Atem- und Bewegungstherapie sowie mentales Gehtraining.

**Gruppe A: Konventionelle Kur:** Bei Lungenhochdruck dürfen die Patienten in der Regel bei der konventionellen Kur keine körperlichen Belastungen vornehmen. Die Therapie besteht hier in gesunder Ernährung, körperlicher Schonung, Massagen, Krankengymnastik und leichten Spaziergängen. Diese Maßnahmen, insbesondere die körperliche Schonung, werden derzeit allgemein für Patienten mit Lungenhochdruck empfohlen.

**Gruppe B: Konventionelle Kur + Atem- und Bewegungstherapie + mentales Gehtraining:** Diese Rehabilitationsmaßnahme enthält alle Elemente der konventionellen Kur wird aber an Stelle der körperlichen Schonung durch eine vorsichtige Atem- und Bewegungstherapie ergänzt. Zudem wird ein mentales Gehtraining durchgeführt, bei dem die Patienten u.a. lernen sollen, Bewegungsabläufe kraftsparender durchzuführen.

Die beiden Gruppen werden gebildet, da man derzeit nicht sicher weiß, ob die Atem-

und Bewegungstherapie überhaupt sinnvoll ist und nicht sogar schaden kann. Zudem soll untersucht werden, ob durch einen „Erholungsurlaub“ allein (Gruppe A) nicht auch schon eine deutliche Verbesserung erreicht werden kann. Die Zugehörigkeit zu den Gruppen wird nach der zusage durch den Kostenträger per Zufall (Los) ermittelt, damit niemand benachteiligt ist. Die Teilnehmer, die dennoch eine Atem- und Bewegungstherapie durchführen möchten, können nach 3 Monaten (auf unsere Kosten) nochmals eine 3-wöchige Kur erhalten, bei der dann die Atem- und Bewegungstherapie durchgeführt wird.

**Teilnahme:** Wer kann bei dieser Reha-Maßnahme teilnehmen? Alle Patienten, mit einem chronischem Lungenhochdruck, die seit mindestens 3 Monaten unter optimierter medikamentöser Therapie gut und stabil eingestellt sind. Dies ist wichtig, da die geplanten Maßnahmen, die medikamentöse Therapie ergänzen sollen und die Medikamente während der Kur sowie 3 Monate danach möglichst unverändert beibehalten werden sollen. Ansonsten weiß man bei Änderungen der Gehstrecke z.B. nicht, ob diese durch die Änderung der Medikation oder durch die Kurmaßnahmen verursacht wurden. Die Teilnahme an diesem Rehabilitationsprojekt ist freiwillig und kann jederzeit beendet werden.

**Ziele der Atem- und Bewegungstherapie sind:**

1) die Symptome zu lindern

2) die Lebensqualität zu verbessern

3) die Leistungsfähigkeit zu verbessern

4) eine spezielle Atemtherapie für die pulmonale Hypertonie zu entwickeln

**Vorgesehene Untersuchungen:**

Zu Beginn der Kur, an deren Ende sowie 3 Monaten später soll eine klinische Kontrolluntersuchung durchgeführt werden, mit deren Hilfe die Intensität der Trainingseinheit als auch der Erfolg der Atem- und Bewegungstherapie überprüft werden soll. Zu den Untersuchungen zählen ein Fragebogen zu Ihrer Lebensqualität, die körperliche Untersuchung, EKG und Herzultraschall in Ruhe und unter Belastung, Lungenfunktionsuntersuchung in Ruhe und unter Belastung, ein 6-Minuten Gehtest, Laboruntersuchungen sowie eine Magnetresonanztomographie.

**Nachuntersuchungen:**

Die Therapie soll nach der Rehabilitation ambulant weitergeführt werden. Nach 3 Monaten findet dann ein Nachuntersuchung in der Universitätsklinik Heidelberg statt. Dabei werden die gleichen Verfahren, wie bei der Eingangsuntersuchung verwendet. Für die Nachuntersuchung können Sie kostenlos für 2 Tage in der Rehabilitationsklinik Königsstuhl übernachten.

**Risiken:** Bei Patienten mit Linksherzinsuffizienz hat sich gezeigt, dass die Bewegungstherapie zwar sehr effektiv ist, aber eine Gratwanderung darstellt. Trainiert man zuviel, können Komplikationen auftreten. Bei zu wenig intensivem Training, tritt oft nicht der gewünschte Trainingseffekt ein. Komplikationen bei Überanstrengung können sein, dass die Gefäßveränderungen in der Lunge zunehmen und sich der Lungenhochdruck weiter verschlechtert. Dies kann das rechte Herz weiter belasten und zu einer Rechtsherzdekompensation führen, bis hin zum plötzlichen Herztod. Auch können gefährliche Rhythmusstörungen auftreten. Die Übungen müssen daher genau auf Ihr persönliches, körperliches Leistungsvermögen angepasst werden und es ist sehr wichtig, dass Sie während der Kur auf mögliche Frühzeichen der Überforderung achten.

**Chancen:** Andererseits bietet das körperliche Training möglicherweise die Chance, Ihre körperliche Leistungsfähigkeit zu steigern und dadurch mehr Lebensqualität zu erreichen. Zudem könnte es den Krankheitsverlauf positiv beeinflussen.

**Datenschutz**

Sämtliche bei den medizinischen Untersuchungen und der Bewegungs- und Atemtherapie erhaltenen Daten werden anonymisiert und sind Dritten nicht zugänglich. Die Vorschriften über die ärztliche Schweigepflicht und den Datenschutz werden gewahrt.

**Rücktritt:** Die Teilnahme an diesem Rehabilitationsprojektes ist freiwillig. Jeder, der an dieser Studie teilnimmt, kann zu jedem Zeitpunkt seine Mitarbeit beenden und die Löschung seiner Daten verlangen.

Heidelberg, den 29.08.26 PD Dr. med. Ekkehard Grünig

**Merkblatt mit Übungen für zu Hause:**

Für alle Übungen gilt:

- jeder übt so gut er kann
- immer eine kurze Pause zwischen den einzelnen Übungen und immer wieder die Wahrnehmung der Atembewegung einschieben
- nicht so viele Wiederholungen am Stück, lieber mehrere Serien
- 3-5mal die Woche üben, ca.20 min., 3-5 Übungen aus unterschiedlichen Bereichen auswählen ( z.B. Wahrnehmung, Mobilisation und Entspannung)

**Übungsbeispiele:**

***1.Wahrnehmung der Atembewegung in Rückenlage, Bauchlage, Sitz und Stand:***

Hände auf das Brustbein, Bauch, Rücken oder seitlich an die Rippen legen. Versuchen dorthin zu atmen, wo man die Hände spürt, gegen die Hände atmen.

Diese Übung mit der dosierten Lippenbremse kombinieren!

**Ziele:** Wahrnehmung, Entspannung, Atemvertiefung

**Zusatz:** Erfahrbares Atmen nach Middendorf;

sanfter Druck zweier Fingerkuppen aneinander, eher seitlich; z.B. der Zeigefinger

oder Mittelfinger, auch mal alle Fingerkuppen aneinander legen;

spüren, wo die Atembewegung stattfindet;

***2. Einatemtechniken:***

- Tief einatmen und dann Luft ca. 10 Sekunden anhalten, mit Lippenbremse ausatmen
- Bei der Einatmung so oft es geht schnüffeln, Luft kurz anhalten und dann langsam, z.B. mit der Lippenbremse ausatmen.
- Nasenstenose:

1. mit Finger ein Nasenloch zuhalten, tief einatmen und durch Mund,

evtl. mit Lippenbremse, ausatmen

1. beide Nasenlöcher mit den Fingerkuppen verengen, dann wie bei einem

***3. Dehnlagen/ Mobilisation:***

Während der Dehnung versuchen in den gedehnten Bereich hinein zu atmen.

Wenn die Spannung an der Thoraxseite zu stark ist, mit einer Hand den Packegriff machen, über paar Atemzüge halten und dann lösen. Wenn die Spannung dann nachgelassen hat, Arm wieder in die Dehnung bringen!

- **Drehdehnlage**: Rückenlage, Beine anstellen, Knie aneinander, Arme hinter den Kopf

nehmen, und dann Beine auf eine Seite fallen lassen. Die Schultern sollen dabei auf dem Boden bleiben und die Knie sollen genau übereinander liegen. Gegebenenfalls die Arme oder Beine mit einem Kissen unterlagern lassen. Dehnlage mind. 2-3 Minuten beibehalten und dann langsam auflösen. Nach einer kurzen Pause andere Seite.

Varianten:

1. Diese Dehnlage kann auch von der Seitlage aus begonnen werden. Dabei das obere Bein anbeugen und das untere strecken, oder beide Beine anbeugen und Knie über einander legen, so dass die Ausgangsstellung stabil ist. Dann die Hand des obenliegendem Arm hinter den Kopf nehmen und den Ellbogen nach hinten führen/ fallenlassen. Mit dem Kopf nachschauen.
2. Seitlage: beide Beine anbeugen

Den oberen Arm gestreckt bei der Einatmung nach hinten oben führen und bei der Ausatmung wieder zurücknehmen, mit Lippenbremse kombinieren; die Knie beleiben immer zusammen und liegen übereinander!

1. Rückenlage: ein Bein anbeugen und mit der gegenüberliegenden Hand von außen am Knie greifen; dann zur Seite ziehen und Arm der gleichen Seite abspreizen

**- Streckdehnung**: Rückenlage:

Mit dem Atemrhythmus einen Arm nach oben strecken und gleichzeitig die Ferse der selben Seite nach

unten raus schieben. Während der Ausatmung Anspannung wieder lösen.

Immer ein Atemzug Pause dazwischen!

**Variante:**

1. Seitlage, unteres Bein angebeugt, Kopf unterlagern mit Kissen oder Arm: wie in Rückenlage Arm nach oben und Bein nach unten rausschieben, ein Atemzug Pause
2. Seitlage: Streckdehnlage mit Bewegung, d.h. mit der Ausatmung Ellbogen oder Hand und Knie zusammenbringen; nur ca. 5 Wiederholungen am Stück und dann Pause!

- **C – Lage / Halbmondlage**: Rückenlage

Einen Arm über den Kopf legen, so dass, wenn möglich, der Ellbogen auf der Unterlage

liegt. Wenn Schmerzen in der Schulter entstehen, Arm mit Kissen unterlagern. Dann das

entgegengesetzte Bein anspannen, Fuß hochziehen und gestreckt nach außen führen,

ablegen und lösen.

Mit dem Arm der gleichen Seite über die Unterlage nun Richtung Knie „krabbeln“,

**Ziele der Dehnlagen**: - Entspannung

- Verbesserung der Ventilation

- Herabsetzung erhöhter Gewebswiderstände in der Haut und in der

Muskulatur

- verstärkte Atembewegung im gedehnten Bereich

**Weitere Dehnungen**:

- Dehnung der Adduktoren/ Oberschenkelinnenseite:

4-Füßler-Stand, Knie soweit wie möglich auseinander, auf sie Unterarme stützen;

mit der Einatmung das Gesäß Richtung Fersen bringen, so daß eine Dehnung spürbar ist; bei der Ausatmung langsam wieder aus der Dehnung rausgehen; paar Wiederholungen

- Dehnung der Hüftflexoren:

Aus dem Kniestand ein Bein nach vorne aufstellen, ca. 90° im

Kniegelenk, für mehr Stabilität mit einer Hand an einem Stuhl oder Ähnlichem festhalten;

Mit der Einatmung das Becken nach vorne schieben und bei der Ausatmung

wieder zurück.

- Drehsitz: im Langsitz ein Bein anstellen, mit beiden Händen das Knie umfassen und bei der Ausatmung aus der Wirbelsäule nach oben dehnen; bei der Ausatmung zur Seite des angestellten Beines drehen; einatmend mit der Außenseite des gegenüberliegenden Ellbogens gegen die Außenseite des Knies drücken; die andere Hand hinter dem Gesäß aufstellen; dann die Schultern entspannen und diese Position über einige Atemzüge halten; Entspannung: beide Beine anstellen, umfassen und Kopf auf den Knien ablegen

Der Drehsitz ist auch auf einem Hocker oder Stuhl durchzuführen!

- Aufrechte Lagerung: Rückenlage, Arme nach oben nehmen, gegebenenfalls unterlagern;

Beine etwas auseinander, in den Bauch atmen, 5-10min so liegen beleiben, Position soll

angenehm sein

**4. *Sekretolyse:***

- **Autogene Drainage:**

Sitz mit angelehntem Rücken: eine Hand zur Wahrnehmung auf die Brust legen;

Zuerst von der normalen Atmung immer tiefer werdende Atemzüge durch die Nase ausführen. Nach dem Einatmen Luft immer kurz anhalten. Zunächst geschieht dann die Ausatmung passiv, aber dann versuchen lange auszuatmen mit sanftem Einsatz der Ausatemmuskulatur.

Beispiel: **Einatmen – Luft kurz anhalten – ausatmen – lange ausatmen**

Das wird so oft wiederholt, bis das Sekret unter der Hand spürbar wird und als Rasseln hörbar. Dann kann es mit 1-2 Hustenstößen abgehustet werden.

Es bietet sich an diese Übung mit der Lippenbremse zu kombinieren!

Diese Technik eignet sich vor allem bei sehr starker Verschleimung!

- **Schnüffeln:**

Bei der Einatmung so lang es geht schnüffeln, Luft kurz anhalten und dann langsam, z.B.

mit der Lippenbremse ausatmen.

***5. Übungen aus dem Yoga:***

Als Entspannungspositionen bei den Yogaübungen eignen sich der Päckchen-,

Kutschersitz oder einfach die Rücken- bzw. Bauchlage.

Für alle Übungen gilt hier besonders: Jeder führt die Übungen so lang durch, wie sie im

angenehm sind! Wenn eine Übung zu anstrengend wird, aufhören!

***-*  Krokodil :** Rückenlage

Arme auf Schulterhöhe abgespreizt und gestreckt hinlegen, mit dem Handrücken leicht auf

die Unterlage drücken; mit der Achillessehne zwischen den 1. und 2. Zeh des anderen

Fußes legen; Becken zur Seite kippen ( wenn rechter Fuß oben, nach links kippen) und so

lang in dieser Stellung bleiben, wie der Körper es selbst will, Kopf in die andere Richtung

drehen.

**Varianten des Krokodils**:

- Rückenlage: Arme auf Schulterhöhe abgespreizt und gestreckt hinlegen, mit dem

Handrücken leicht auf die Unterlage drücken, rechts Fußspitze hochziehen,

Bein anspannen und bei der Einatmung gestreckt abheben, gestreckt auf der linken Seite

so hoch wie möglich mit der Ausatmung ablegen (versuchen mit der Fußspitze die Hand

zu berühren), Kopf nach links drehen.

Die Schultern sollen dabei immer auf dem Boden bleiben, lieber das Bein nicht so hoch

ablegen!

Stellung 2-3 Minuten halten, Ausgangsstellung langsam wieder einnehmen, kurze Pause

und dann die andere Seite.

- Ausgangsstellung wie oben, Beine weit auseinander anstellen, Knie zur Seite fallen

lassen und versuchen mit den Knien den Boden zu berühren, Schultern bleiben auf dem

Boden

- **Die Katze**:

Vierfüßlerstand: bei der Einatmung den Kopf nach oben nehmen und Rücken

durchhängen lassen;

bei der Ausatmung Wirbelsäule rund machen, Kopf mitnehmen

- **Der Fisch:**

Rückenlage: Arme dicht an den Körper nehmen; dann auf die Ellenbogen stützen und das

Brustbein so weit es geht nach oben nehmen, ruhig weiteratmen, über ein paar Atemzüge

halten

**Ziele:** Yogaübungen haben viele verschieden Wirkungen. Die Wirkungen, die uns hierbei

interessieren sind:

- Thoraxmobilisation
- Entspannung
- Kräftigung der aufrichtenden Muskulatur
- Dehnung verschiedener Thoraxabschnitte und Muskulatur
- Atemreize für verschiedene Thoraxabschnitte
- der ganze Körper wird mit einbezogen

***6. Gymnastik auf dem Hocker (oder Pezziball):***

a) **ohne Gerät:**

- ganz gerade hinsetzen und dann so weit zusammenrollen wie möglich (Becken

kippen, nicht Oberkörper nach vorne und hinten neigen!).Mit Atmung kombinieren

(Einatmung aufrichten und Ausatmung zusammenrollen).

- Hände hinter dem Kopf verschränken, Ellenbogen soweit wie möglich parallel zu den

Schultern halten. Bei der Einatmung aufrichten und Ellenbogen so weit wie möglich nach

hinten führen, bei der Ausatmung zusammenrollen und Ellenbogen vor dem Körper

zusammenführen.

- einen Arm gestreckt über die Seite nach oben nehmen, abwinkeln und über den Kopf das

Ohr der anderen Seite berühren, mit der anderen Hand an der Brustkorbseite

Atembewegung spüren

- beide Hände ruhen auf einem Knie z. B. links. Den rechten Arm gestreckt nach hinten

oben seitlich nehmen und der Hand nachschauen. Die Schultern sollen dabei möglichst

nach vorne zeigen! Eine Seite paar Mal wiederholen und dann Wechsel.

- Hände wieder hinter dem Kopf verschränken und dann den Oberkörper leicht nach rechts

und links neigen. In der gleichen Ebene bleiben!

- Ausgangsstellung wie bei der Übungen gerade eben. Oberkörper nach rechts und links

drehen.

- Schultern hochziehen und fallen lassen, dann auch Schultern nach vorne und hinten

kreisen.

- Wahrnehmung der Atembewegung ( Bauch, Rippen, Brustbein, Rücken)

b) **zum Beispiel mit dem Handtuch:**

- aufrechter Sitz, Handtuch zu einer „Wurst“/“Schlange“ falten

- Handtuch um den Brustkorb legen, Enden festhalten, Zusammenziehen beim Ausatmen

und Richtungswiderstand beim Einatmen. Aber Bewegung zulassen!

- Handtuch straff an den Bauch legen, Wahrnehmung der Atembewegung gegen das

Handtuch

- Handtuch langsam seitlich auf Schulterhöhe nach hinten führen, hinterer Arm bleibt

gestreckt und Schultern zeigen weiterhin nach vorne! Hinten kurz ziehen und

dann wieder langsam nach vorne. Andere Seite!

- Handtuch über Schulter legen, mit der Hand der gleichen Seite vorne mit der anderen

Hand hinten fassen; Kopf zur Gegenseite neigen („Ohrläppchen Richtung Schulter“) und

Schulter sanft mit dem Handtuch nach unten ziehen; Dehnung kurz halten

- vorstellen, Handtuch wäre ein Paddel! Links und rechts am Körper vorbei paddeln.

Tief „eintauchen“!

Alle Bewegungen mit der Atmung kombinieren, nicht die Luft anhalten und nicht pressen!

Bewegungen langsam ausführen, aber nicht zu langsam oder in einer Stellung zu lange verharren!

Beide Seiten immer gleich beüben!

Es können auch Stäbe, Keulen, Seile, Bohnensäckchen, Bälle und sonstige Gymnastikgeräte eingesetzt werden!

**Zusatzübung bei Gymnastik mit dem Ball:**

- Ball in eine Hand nehmen, Arm gestreckt über die Seite nach oben führen, über dem Kopf

übergeben und auf der anderen Seite mit gestrecktem Arm nach unten führen; evtl. Ball

unter den Beinen durchgeben, oder wieder zurück auf gleicher Seite;

- wenn der Ball klein ist, seitlich unter den Arm legen und Atembewegung spüren

- mit Igelball Körper abrollen

***7. Atemerleichternde Stellungen:***

- **Kutschersitz**: Sitz mit leicht gegrätschten Beinen, Unterarme auf Oberschenkel

ablegen

- **Päckchensitz**: auf den Fersen sitzen und Kopf auf den Unterarmen oder Händen

ablegen

- **Sitz am Tisch**: wie Kutschersitz, nur Arme auf dem Tisch ablegen

- **Torwartstellung:** mit leicht gegrätschten Beinen hinstellen und mit den Händen am

Oberschenkel aufstützen

**- Stand an der Wand/ Joe cool:** mir einer Hand an der Wand abstützen, andere Hand

an die Hüfte

**! ☺ Viel Spaß mit diesen Übungen !**

**1. Patient Interview/Medical History**

**Date** (dd/mm/yy)**:** [ ] [ ] / [ ] [ ] / [ ] [ ]

**1. Patient Interview/Medical History**

**1.1 Demographic Data:**

Height [cm]: [ ] [ ] [ ]

Weight [kg]: [ ] [ ] [ ]

Gender (please tick): male [ ] female [ ]

**1.2 Medical History:**

#### Pulmonary diseases (refer to lists 1.-3.)

Respiratory failure yes [ ] no [ ] If yes, please specify…

______________________________

______________________________

COPD yes [ ] no [ ] If yes, please specify…

______________________________

______________________________

Interstitial lung disease yes [ ] no [ ] If yes, please specify…

______________________________

______________________________

Pulmonary embolism yes [ ] no [ ] If yes, please specify…

______________________________

______________________________

#### Heart diseases (refer to lists 1.-3.)

Cardiovascular events yes [ ] no [ ] If yes, please specify…

______________________________

______________________________

Cardiac arrhythmia/ yes [ ] no [ ] If yes, please specify…

AV Block ______________________________

______________________________

Valvular heart disease yes [ ] no [ ] If yes, please specify…

______________________________

______________________________

Hypertrophic cardiomyopathy yes [ ] no [ ] If yes, please specify…

______________________________

______________________________

Angina pectoris yes [ ] no [ ] If yes, please specify…

______________________________

______________________________

**Other** yes [ ] no [ ] If yes, please specify…

______________________________

______________________________

**1.3 Previous and current therapies**

**Current therapies** yes [ ] no [ ] If yes, please specify below.

| **Drug** (preferably generic name) | Daily dose | Start Date | Continuing? (Please tick) |
| --- | --- | --- | --- |
|  |  |  | □ |
|  |  |  | □ |
|  |  |  | □ |
|  |  |  | □ |
|  |  |  | □ |

**Previous therapies** yes [ ] no [ ] If yes, please specify below.

| **Drug** (preferably generic name) | Daily dose | Start Date | Stop date |
| --- | --- | --- | --- |
|  |  |  |  |
|  |  |  |  |
|  |  |  |  |

#### 1.4 Smoking habits

Is the patient smoker: yes [ ] no [ ] If yes, for how long? [yrs]: [ ] [ ]

If no, has the patient ever smoked yes [ ] no [ ]

If yes, for how many years has the patient stopped smoking? [yrs]: [ ] [ ]

Duration of smoking period? [yrs]: [ ] [ ]

# 1.5 Pedigree

Additional information to the pedigree constructed by interviewing the related index patient

# Interview done [ ]

# 2. Baseline examinations at rest

# 2.1 Physical examination at the beginning of the rehabilitation

Blood pressure RRsys [mmHg]: [ ] [ ] [ ] RRdia [mmHg]: [ ] [ ] [ ]

Heart rate [min^-1]^: [ ] [ ] [ ]

Auscultation of heart and lungs:

Heart (please tick): normal [ ] abnormal [ ] If "abnormal" please specify…

____________________________________

____________________________________

Lungs (please tick): normal [ ] abnormal [ ] If "abnormal", please specify…

____________________________________

____________________________________

Other physical examinations (e.g. abdomen) (please tick):

Abdomen normal [ ] abnormal [ ] If "abnormal", please specify…

____________________________________

____________________________________

Other (please specify)

___________________________________________________________________________

___________________________________________________________________________

**2.2 ECG at rest**

Documentation over 30 seconds

(Please tick)

Normal [ ] abnormal [ ] If "abnormal", please specify…

________________________________________________

**2.3** **Echocardiography at rest**

Measurement of heart structures and cavities, assessment of function

| **M-Mode** |  |
| --- | --- |
| Aortic root [mm] | [ ] [ ] |
| Left atrium [mm] | [ ] [ ] |
| Right ventricle [mm] | [ ] [ ] |
| Inferior Vena cava [mm] | [ ] [ ] |
| Interventricular septum [mm] | [ ] [ ] |
| Septal motion  Normal [ ]  abnormal [ ]  If abnormal, please specify in the right column |  |
| Posterior wall [mm] | [ ] [ ] |
| Posterior wall motion  Normal [ ]  abnormal [ ]  If abnormal, please specify in the right column |  |
| LV-EDD [mm] | [ ] [ ] |
| LV-ESD [mm] | [ ] [ ] |
|  |  |
| **2-dimensional examination** |  |
| Aortic valve  Normal [ ]  abnormal [ ]  If abnormal, please specify in the right column |  |
| Mitral valve  Normal [ ]  abnormal [ ]  If abnormal, please specify in the right column |  |

| Tricuspid valve  Normal [ ]  abnormal [ ]  If abnormal, please specify in the right column |  |
| --- | --- |
| Pulmonary valve  Normal [ ]  abnormal [ ]  If abnormal, please specify in the right column |  |
| Right ventricular systolic function  Normal [ ]  abnormal [ ]  If abnormal, please specify in the right column |  |
| Regional wall motion  Normal [ ]  abnormal [ ]  If abnormal, please specify in the right column |  |
| Pericardial effusion | Yes [ ], no [ ] |
|  |  |
| **Doppler examination** |  |
| Aortic valve [ms^-1^] | [ ] , [ ] |
| Mitral valve [ms^-1^] | [ ] , [ ] |
| Tricuspid valve [ms^-1^] | [ ] , [ ] |
| Tricuspid regurgitation velocity [ms^-1^] | [ ] , [ ] [ ] |
| Pulmonary valve [ms^-1^] | [ ] , [ ] |
| RVOT, AT [ms] | [ ] [ ] [ ] |
| RVOT, ET [ms] | [ ] [ ] [ ] |
| Right ventricular contraction time [ms] | [ ] [ ] [ ] |
| TAPSE [mm] | [ ] [ ] |

General assessment of RV/LV function (systolic/diastolic)

(please tick) normal [ ] abnormal [ ]

If "abnormal", please specify…

___________________________________________________________________________

___________________________________________________________________________

General assessment of valvular diseases

(please tick) yes [ ] no [ ]

If "yes" please specify…

___________________________________________________________________________

___________________________________________________________________________

# 2.4 Stress Echocardiography / Exercise Echocardiography

**Date of examination** (dd/mm/yy)**:** [ ] [ ] / [ ] [ ] / [ ] [ ]

**Investigator´s name** (in capital letters)**:_____________________________________________**

**Technical Equipment:__________________________________________________________**

# Direct measurements during Stress Echocardiography

| Work-load  [W] | TR jet [ms^-1^] | RV/LV function  Please tick | | O_2_ sat  [%] | BP Sys/dia  [mmHg] | | HR  [min^-1^] | ECG Please tick | | VO_2_  [%] |
| --- | --- | --- | --- | --- | --- | --- | --- | --- | --- | --- |
|  |  | n | a |  | sys | dia |  | n | a |  |
| 0 | [ ],[ ] [ ] |  |  |  |  |  |  |  |  |  |
| 25 | [ ],[ ] [ ] |  |  |  |  |  |  |  |  |  |
| 50 | [ ],[ ] [ ] |  |  |  |  |  |  |  |  |  |
| 75 | [ ],[ ] [ ] |  |  |  |  |  |  |  |  |  |
| 100 | [ ],[ ] [ ] |  |  |  |  |  |  |  |  |  |
| 125 | [ ],[ ] [ ] |  |  |  |  |  |  |  |  |  |
| 150 | [ ],[ ] [ ] |  |  |  |  |  |  |  |  |  |
| 175 | [ ],[ ] [ ] |  |  |  |  |  |  |  |  |  |
| 200 | [ ],[ ] [ ] |  |  |  |  |  |  |  |  |  |
| post | [ ],[ ] [ ] |  |  |  |  |  |  |  |  |  |

RV/LV function and ECG: n=normal, a=abnormal

If abnormal, please specify giving the workload at which abnormalities occurred (e.g. RV/LV (75 W): …):

RV/LV: ___________________________________________________________________________________

__________________________________________________________________________________________

ECG: _____________________________________________________________________________________

__________________________________________________________________________________________

**TR jet at maximum exercise** [ms^-1^]**:** [ ], [ ] [ ]

**worklord** at which examination was terminated [W]: [ ] [ ] [ ]

Technical difficulties during the examination

Please specify:

__________________________________________________________________________________________

__________________________________________________________________________________________

__________________________________________________________________________________________

**Patient Interview**

**Reasons** for terminating exercise

Please instruct the patient to place a mark on the scale according to his/her reasons for breaking off exercise. (The distance from the left end point will be measured at the coordinating centre.)

*I stopped due to shortness of breath.*

**I**____I____I____I____I____**I**____I____I____I____I____**I**

not true at all absolutely true [ ] [ ] [ ] mm

(to be filled by coord.)

###### I stopped due to chest pain.

**I**____I____I____I____I____**I**____I____I____I____I____**I**

not true at all absolutely true [ ] [ ] [ ] mm

(to be filled by coord.)

###### I stopped due to leg pain/exhaustion.

**I**____I____I____I____I____**I**____I____I____I____I____**I**

not true at all absolutely true [ ] [ ] [ ] mm

(to be filled by coord.)

###### I stopped due to general exhaustion.

**I**____I____I____I____I____**I**____I____I____I____I____**I**

not true at all absolutely true [ ] [ ] [ ] mm

(to be filled by coord.)

###### I stopped due to other reasons.

**I**____I____I____I____I____**I**____I____I____I____I____**I**

not true at all absolutely true [ ] [ ] [ ] mm

(to be filled by coord.)

**2.5 Spiroergometry**

**Date of examination** (dd/mm/yy)**:** [ ] [ ] / [ ] [ ] / [ ] [ ]

**Investigator´s name** (in capital letters)**:**

**_____________________________________________**

**Technical equipment:**

**___________________________________________________________________________**

Continuous measurements:

O_2_ uptake (VO_2_) End-tidal fraction of O_2_ (FETO_2_)

CO_2_ exhalation (VCO_2_) End-tidal fraction of CO_2_ (FETCO_2_)

Tidal volume (VT) Partial pressure of end-expiratory O_2_ (PETO_2_)

Ventilation (VE) Respiratory quotient (RQ)

Breathing frequency (BF) Ventilatory equivalent (EQO_2_)

Mixed expiratory fraction of O_2_ (FO_2_) Heart rate (HR)

Mixed expiratory fraction of CO_2_ (FCO_2_) Blood pressure (BP)

12 lead ECG

NB: It will facilitate recording of the parameters if you modify the programming of your spiroergo device to give all the readouts in the table below in print.

**Measurements Spiroergometry**

| Work-load  [W] | **VO_2_**  [l/min] | **VCO_2_**  [l/min] | **V** [l] | **VE**  [l/min] | **BF** [min^-1^] | **FO_2_**  [%] | **FCO_2_**  [%] | **FETO_2_**  [%] |
| --- | --- | --- | --- | --- | --- | --- | --- | --- |
| 0 |  |  |  |  |  |  |  |  |
| 25 |  |  |  |  |  |  |  |  |
| 50 |  |  |  |  |  |  |  |  |
| 75 |  |  |  |  |  |  |  |  |
| 100 |  |  |  |  |  |  |  |  |
| 125 |  |  |  |  |  |  |  |  |
| 150 |  |  |  |  |  |  |  |  |
| 175 |  |  |  |  |  |  |  |  |
| 200 |  |  |  |  |  |  |  |  |
| post |  |  |  |  |  |  |  |  |
| AT |  |  |  |  |  |  |  |  |

AT= anaerobic threshold Table continues on the next page.

**Measurements Spiroergometry** (cont.)

| Work-load  [W] | **FETCO_2_** **[**%] | **PETO_2_** [mmHg] | **RQ** | **BP** [mmHg] | | **HR**  [min^-1^] | **ECG** Please tick | |
| --- | --- | --- | --- | --- | --- | --- | --- | --- |
|  |  |  |  | sys | dia |  | n | a |
| 0 |  |  |  |  |  |  |  |  |
| 25 |  |  |  |  |  |  |  |  |
| 50 |  |  |  |  |  |  |  |  |
| 75 |  |  |  |  |  |  |  |  |
| 100 |  |  |  |  |  |  |  |  |
| 125 |  |  |  |  |  |  |  |  |
| 150 |  |  |  |  |  |  |  |  |
| 175 |  |  |  |  |  |  |  |  |
| 200 |  |  |  |  |  |  |  |  |
| post |  |  |  |  |  |  |  |  |
| AT |  |  |  |  |  |  |  |  |

ECG: n=normal, a=abnormal

If abnormal, please specify giving workloads (e.g. ECG (75 W):….)

___________________________________________________________________________

___________________________________________________________________________

**Workload** at which examination was terminated [W]: [ ] [ ] [ ]

# Technical difficulties during the examination

Please specify:

_____________________________________________________________________________

_____________________________________________________________________________

_____________________________________________________________________________

# 3. Baseline examinations at rest

# 3.1 Physical examination after 3 weeks

Blood pressure RRsys [mmHg]: [ ] [ ] [ ] RRdia [mmHg]: [ ] [ ] [ ]

Heart rate [min^-1]^: [ ] [ ] [ ]

Auscultation of heart and lungs:

Heart (please tick): normal [ ] abnormal [ ] If "abnormal" please specify…

____________________________________

____________________________________

Lungs (please tick): normal [ ] abnormal [ ] If "abnormal", please specify…

____________________________________

____________________________________

Other physical examinations (e.g. abdomen) (please tick):

Abdomen normal [ ] abnormal [ ] If "abnormal", please specify…

____________________________________

____________________________________

Other (please specify)

___________________________________________________________________________

___________________________________________________________________________

**3.2 ECG at rest**

Documentation over 30 seconds

(Please tick)

Normal [ ] abnormal [ ] If "abnormal", please specify…

________________________________________________

**3.3** **Echocardiography at rest**

Measurement of heart structures and cavities, assessment of function

| **M-Mode** |  |
| --- | --- |
| Aortic root [mm] | [ ] [ ] |
| Left atrium [mm] | [ ] [ ] |
| Right ventricle [mm] | [ ] [ ] |
| Inferior Vena cava [mm] | [ ] [ ] |
| Interventricular septum [mm] | [ ] [ ] |
| Septal motion  Normal [ ]  abnormal [ ]  If abnormal, please specify in the right column |  |
| Posterior wall [mm] | [ ] [ ] |
| Posterior wall motion  Normal [ ]  abnormal [ ]  If abnormal, please specify in the right column |  |
| LV-EDD [mm] | [ ] [ ] |
| LV-ESD [mm] | [ ] [ ] |
|  |  |
| **2-dimensional examination** |  |
| Aortic valve  Normal [ ]  abnormal [ ]  If abnormal, please specify in the right column |  |
| Mitral valve  Normal [ ]  abnormal [ ]  If abnormal, please specify in the right column |  |

| Tricuspid valve  Normal [ ]  abnormal [ ]  If abnormal, please specify in the right column |  |
| --- | --- |
| Pulmonary valve  Normal [ ]  abnormal [ ]  If abnormal, please specify in the right column |  |
| Right ventricular systolic function  Normal [ ]  abnormal [ ]  If abnormal, please specify in the right column |  |
| Regional wall motion  Normal [ ]  abnormal [ ]  If abnormal, please specify in the right column |  |
| Pericardial effusion | Yes [ ], no [ ] |
|  |  |
| **Doppler examination** |  |
| Aortic valve [ms^-1^] | [ ] , [ ] |
| Mitral valve [ms^-1^] | [ ] , [ ] |
| Tricuspid valve [ms^-1^] | [ ] , [ ] |
| Tricuspid regurgitation velocity [ms^-1^] | [ ] , [ ] [ ] |
| Pulmonary valve [ms^-1^] | [ ] , [ ] |
| RVOT, AT [ms] | [ ] [ ] [ ] |
| RVOT, ET [ms] | [ ] [ ] [ ] |
| Right ventricular contraction time [ms] | [ ] [ ] [ ] |
| TAPSE [mm] | [ ] [ ] |

General assessment of RV/LV function (systolic/diastolic)

(please tick) normal [ ] abnormal [ ]

If "abnormal", please specify…

___________________________________________________________________________

___________________________________________________________________________

General assessment of valvular diseases

(please tick) yes [ ] no [ ]

If "yes" please specify…

___________________________________________________________________________

___________________________________________________________________________

# 3.4 Stress Echocardiography / Exercise Echocardiography

**Date of examination** (dd/mm/yy)**:** [ ] [ ] / [ ] [ ] / [ ] [ ]

**Investigator´s name** (in capital letters)**:_____________________________________________**

**Technical Equipment:__________________________________________________________**

# Direct measurements during Stress Echocardiography

| Work-load  [W] | TR jet [ms^-1^] | RV/LV function  Please tick | | O_2_ sat  [%] | BP Sys/dia  [mmHg] | | HR  [min^-1^] | ECG Please tick | | VO_2_  [%] |
| --- | --- | --- | --- | --- | --- | --- | --- | --- | --- | --- |
|  |  | n | a |  | sys | dia |  | n | a |  |
| 0 | [ ],[ ] [ ] |  |  |  |  |  |  |  |  |  |
| 25 | [ ],[ ] [ ] |  |  |  |  |  |  |  |  |  |
| 50 | [ ],[ ] [ ] |  |  |  |  |  |  |  |  |  |
| 75 | [ ],[ ] [ ] |  |  |  |  |  |  |  |  |  |
| 100 | [ ],[ ] [ ] |  |  |  |  |  |  |  |  |  |
| 125 | [ ],[ ] [ ] |  |  |  |  |  |  |  |  |  |
| 150 | [ ],[ ] [ ] |  |  |  |  |  |  |  |  |  |
| 175 | [ ],[ ] [ ] |  |  |  |  |  |  |  |  |  |
| 200 | [ ],[ ] [ ] |  |  |  |  |  |  |  |  |  |
| post | [ ],[ ] [ ] |  |  |  |  |  |  |  |  |  |

RV/LV function and ECG: n=normal, a=abnormal

If abnormal, please specify giving the workload at which abnormalities occurred (e.g. RV/LV (75 W): …):

RV/LV: ___________________________________________________________________________________

__________________________________________________________________________________________

ECG: _____________________________________________________________________________________

__________________________________________________________________________________________

**TR jet at maximum exercise** [ms^-1^]**:** [ ], [ ] [ ]

**worklord** at which examination was terminated [W]: [ ] [ ] [ ]

Technical difficulties during the examination

Please specify:

__________________________________________________________________________________________

__________________________________________________________________________________________

__________________________________________________________________________________________

**Patient Interview**

**Reasons** for terminating exercise

Please instruct the patient to place a mark on the scale according to his/her reasons for breaking off exercise. (The distance from the left end point will be measured at the coordinating centre.)

*I stopped due to shortness of breath.*

**I**____I____I____I____I____**I**____I____I____I____I____**I**

not true at all absolutely true [ ] [ ] [ ] mm

(to be filled by coord.)

###### I stopped due to chest pain.

**I**____I____I____I____I____**I**____I____I____I____I____**I**

not true at all absolutely true [ ] [ ] [ ] mm

(to be filled by coord.)

###### I stopped due to leg pain/exhaustion.

**I**____I____I____I____I____**I**____I____I____I____I____**I**

not true at all absolutely true [ ] [ ] [ ] mm

(to be filled by coord.)

###### I stopped due to general exhaustion.

**I**____I____I____I____I____**I**____I____I____I____I____**I**

not true at all absolutely true [ ] [ ] [ ] mm

(to be filled by coord.)

###### I stopped due to other reasons.

**I**____I____I____I____I____**I**____I____I____I____I____**I**

not true at all absolutely true [ ] [ ] [ ] mm

(to be filled by coord.)

**3.5 Spiroergometry**

**Date of examination** (dd/mm/yy)**:** [ ] [ ] / [ ] [ ] / [ ] [ ]

**Investigator´s name** (in capital letters)**:**

**_____________________________________________**

**Technical equipment:**

**___________________________________________________________________________**

Continuous measurements:

O_2_ uptake (VO_2_) End-tidal fraction of O_2_ (FETO_2_)

CO_2_ exhalation (VCO_2_) End-tidal fraction of CO_2_ (FETCO_2_)

Tidal volume (VT) Partial pressure of end-expiratory O_2_ (PETO_2_)

Ventilation (VE) Respiratory quotient (RQ)

Breathing frequency (BF) Ventilatory equivalent (EQO_2_)

Mixed expiratory fraction of O_2_ (FO_2_) Heart rate (HR)

Mixed expiratory fraction of CO_2_ (FCO_2_) Blood pressure (BP)

12 lead ECG

NB: It will facilitate recording of the parameters if you modify the programming of your spiroergo device to give all the readouts in the table below in print.

**Measurements Spiroergometry**

| Work-load  [W] | **VO_2_**  [l/min] | **VCO_2_**  [l/min] | **V** [l] | **VE**  [l/min] | **BF** [min^-1^] | **FO_2_**  [%] | **FCO_2_**  [%] | **FETO_2_**  [%] |
| --- | --- | --- | --- | --- | --- | --- | --- | --- |
| 0 |  |  |  |  |  |  |  |  |
| 25 |  |  |  |  |  |  |  |  |
| 50 |  |  |  |  |  |  |  |  |
| 75 |  |  |  |  |  |  |  |  |
| 100 |  |  |  |  |  |  |  |  |
| 125 |  |  |  |  |  |  |  |  |
| 150 |  |  |  |  |  |  |  |  |
| 175 |  |  |  |  |  |  |  |  |
| 200 |  |  |  |  |  |  |  |  |
| post |  |  |  |  |  |  |  |  |
| AT |  |  |  |  |  |  |  |  |

AT= anaerobic threshold Table continues on the next page.

**Measurements Spiroergometry** (cont.)

| Work-load  [W] | **FETCO_2_** **[**%] | **PETO_2_** [mmHg] | **RQ** | **BP** [mmHg] | | **HR**  [min^-1^] | **ECG** Please tick | |
| --- | --- | --- | --- | --- | --- | --- | --- | --- |
|  |  |  |  | sys | dia |  | n | a |
| 0 |  |  |  |  |  |  |  |  |
| 25 |  |  |  |  |  |  |  |  |
| 50 |  |  |  |  |  |  |  |  |
| 75 |  |  |  |  |  |  |  |  |
| 100 |  |  |  |  |  |  |  |  |
| 125 |  |  |  |  |  |  |  |  |
| 150 |  |  |  |  |  |  |  |  |
| 175 |  |  |  |  |  |  |  |  |
| 200 |  |  |  |  |  |  |  |  |
| post |  |  |  |  |  |  |  |  |
| AT |  |  |  |  |  |  |  |  |

ECG: n=normal, a=abnormal

If abnormal, please specify giving workloads (e.g. ECG (75 W):….)

___________________________________________________________________________

___________________________________________________________________________

**Workload** at which examination was terminated [W]: [ ] [ ] [ ]

# Technical difficulties during the examination

Please specify:

_____________________________________________________________________________

_____________________________________________________________________________

_____________________________________________________________________________

# 4. Exclusion Criteria

| Exclusion criteria for exercise (please tick) | **yes** | **no** |
| --- | --- | --- |
| Acute systemic illness, e.g. infections | □ | □ |
| Acute cardiovascular event within the last 6 months (acute coronary syndrome/MI, cerebral ischaemia/stroke) | □ | □ |
| Life-threatening cardiac arrhythmia (LOWN IVb) | □ | □ |
| Advanced AV block | □ | □ |
| Uncontrolled systemic hypertension with blood pressure at rest 180/100 | □ | □ |
| Advanced valvular heart disease, especially aortic stenosis >I° | □ | □ |
| Hypertrophic cardiomyopathy | □ | □ |
| Pulmonary embolism / DVT within the last 6 months | □ | □ |
| Pregnancy | □ | □ |
| Angina pectoris | □ | □ |
| Inability to undergo exercise testing (e.g. knee or hip problems) | □ | □ |

| Criteria pointing at secondary cause of PH | | |
| --- | --- | --- |
|  | **yes** | **no** |
| A. Significant heart diseases |  |  |
| Coronary heart disease (previous MI / acute coronary syndrome, coronary artery stenosis >70 %, typical symptoms of angina | □ | □ |
| Cardiac arrhythmia (atrial fibrillation / flutter, AV-Block >I° at rest | □ | □ |
| Congestive heart failure: previous history of cardiogenic pulmonary oedema, increased size of LA(>50 mm), LVEF <50 %) | □ | □ |
| Cardiomyopathies: history, signs, or established diagnosis | □ | □ |
| Significant valvular heart disease: MVD >I°, AVD >I°, tricuspid/pulmonary stenosis | □ | □ |
| Significant cardiac left-to-right shunts (shunt volumes >20%) | □ | □ |
| Previous closure of systemic pulmonary shunt, heart valve replacement, cardiac transplantation | □ | □ |
| Systemic hypertension during exercise (RR >210/110 mm Hg) | □ | □ |
|  |  |  |
| B. Significant lung diseases |  |  |
| Respiratory failure (with pO_2_ <90 - 1/3age, or pCO_2_ >40 mmHg) | □ | □ |
| Moderate to severe COPD: FEV1/FVC <80% predicted and FEV1<70% predicted | □ | □ |
| Interstitial lung disease: VC <70% predicted; if present CT: signs of fibrosis | □ | □ |
| Pulmonary embolism at any time: history, clinical signs or established diagnosis | □ | □ |

| C. Other |  |  |
| --- | --- | --- |
| Hepatic cirrhosis: history, clinical signs or established diagnosis | □ | □ |
| HIV infection at any stage: history, clinical signs or established diagnosis | □ | □ |
| History of ingestion of appetite suppressants or drug abuse | □ | □ |
| Severe anaemia (Hb <10 g/dl in female subjects, Hb <12 g/dl in male subjects) | □ | □ |
| Myeloproliferative disorders | □ | □ |
| Sarcoidosis: history, clinical signs or established diagnosis | □ | □ |
| Collagen vascular disease: history, clinical signs or established diagnosis | □ | □ |
| Sleep apnoea syndrome: history, clinical signs or established diagnosis | □ | □ |
| Gaucher disease: history, clinical signs or established diagnosis | □ | □ |
| Schistosomiasis: history, clinical signs or established diagnosis | □ | □ |
| Sickle cell disease: clinical signs or established diagnosis | □ | □ |

## FRAGEBOGEN ZUM GESUNDHEITSZUSTAND

In diesem Fragebogen geht es um Ihre Beurteilung Ihres Gesundheitszustandes **in den letzten 4 Wochen.** Der Bogen ermöglicht es, im Zeitverlauf nachzuvollziehen, wie Sie sich fühlen und wie Sie im Alltag zurechtkommen.

Bitte beantworten Sie jede der folgenden Fragen, in dem Sie bei den Antwortmöglichkeiten die Zahl ankreuzen, die am besten auf Sie zutrifft.

1. **Wie würden Sie Ihren Gesundheitszustand in den letzten 4 Wochen im Allgemeinen beschreiben?**

(Bitte kreuzen Sie nur eine Zahl an)

Ausgezeichnet **. . . . . . . . . . . . . . . . . . . . . . . . . . . . . . . . . . . . .**1

Sehr gut **. . . . . . . . . . . . . . . . . . . . . . . . . . . . . . . . . . . . . . . . . .**2

Gut . **. . . . . . . . . . . . . . . . . . . . . . . . . . . . . . . . . . . . . . . . . . . . .**3

Weniger gut **. . . . . . . . . . . . . . . . . . . . . . . . . . . . . . . . . . . . . . .**4

Schlecht **. . . . . . . . . . . . . . . . . . . . . . . . . . . . . . . . . . . . . . . . . .**5

**2. Im Vergleich zum Jahr davor,** wie würden Sie Ihren

Gesundheitszustand **vor einem Jahr** beschreiben

(Bitte kreuzen Sie nur eine Zahl an)

Derzeit viel besser als vor einem Jahr **. . . . . . . . . . . . . . .** 1

Derzeit etwas besser als vor einem Jahr **. . . . . . . . . . . . .** 2

Etwa so wie vor einem Jahr **. . . . . . . . . . . . . . . . . . . . . . .** 3

Derzeit etwas schlechter als vor einem Jahr **. . . . . . . . . .** 4

Derzeit viel schlechter als vor einem Jahr **. . . . . . . . . . . . .** 5

**3.** Im folgenden sind einige Tätigkeiten beschrieben, die Sie vielleicht an einem normalen Tag ausüben. **Sind sie durch Ihren derzeitigen Gesundheitszustand bei diesen Tätigkeiten eingeschränkt?** Wenn ja, wie stark?

(Bitte kreuzen Sie in jeder Zeile nur eine Zahl an)

| **TÄTIGKEITEN** | **Ja,**  **stark**  **eingeschränkt** | **Ja,**  **etwas**  **eingeschränkt** | **Nein,**  **überhaupt**  **nicht**  **eingeschränkt** |
| --- | --- | --- | --- |
| a. anstrengende Tätigkeiten, z.B. schnell laufen,  schwere Gegenstände heben, anstrengenden  Sport treiben | 1 | 2 | 3 |
| b. mittelschwere Tätigkeiten, z.B. einen Tisch  verschieben, Staub saugen, kegeln, Golf spielen | 1 | 2 | 3 |
| c. Einkaufstaschen heben oder tragen | 1 | 2 | 3 |
| d. mehrer Treppenabsätze steigen | 1 | 2 | 3 |
| e. einen Treppenabsatz steigen | 1 | 2 | 3 |
| f. sich beugen, knien, bücken | 1 | 2 | 3 |
| g. mehr als 1 Kilometer zu Fuß gehen | 1 | 2 | 3 |
| h. mehrere Straßenkreuzungen weit zu Fuß  gehen | 1 | 2 | 3 |
| i. eine Straßenkreuzung weit zu Fuß gehen | 1 | 2 | 3 |
| j. sich baden oder anziehen | 1 | 2 | 3 |

**4.**Hatten Sie in den **vergangenen 4 Wochen aufgrund Ihrer körperlichen Gesundheit** irgendwelche Schwierigkeiten bei der Arbeit oder anderen alltäglichen Tätigkeiten im Beruf bzw. zu Hause?

(Bitte kreuzen Sie in jeder Zeile nur eine Zahl an)

| **SCHWIERIGKEITEN** | **JA** | **NEIN** |
| --- | --- | --- |
| a. Ich konnte nicht so lange wie üblich tätig sein | 1 | 2 |
| b. Ich habe weniger geschafft als ich wollte | 1 | 2 |
| c. Ich konnte nur bestimmte Dinge tun | 1 | 2 |
| d. Ich hatte Schwierigkeiten bei der Ausführung (z.B. ich musste mich  besonders anstrengen) | 1 | 2 |

**5.** Hatten Sie in den **vergangenen 4 Wochen aufgrund seelischer Probleme** irgendwelche Schwierigkeiten bei der Arbeit oder anderen alltäglichen Tätigkeiten im Beruf bzw. zu Hause (z.B. weil Sie sich niedergeschlagen oder ängstlich fühlten)?

(Bitte kreuzen Sie in jeder Zeile nur eine Zahl an)

| **SCHWIERIGKEITEN** | **JA** | **NEIN** |
| --- | --- | --- |
| a. Ich konnte nicht so lange wie üblich tätig sein | 1 | 2 |
| b. Ich habe weniger geschafft als ich wollte | 1 | 2 |
| c. Ich konnte nicht so sorgfältig wie üblich arbeiten | 1 | 2 |

**6.** Wie sehr haben Ihre körperliche Gesundheit oder seelischen Probleme in den **vergangenen 4 Wochen** Ihre normalen Kontakte zu Familienangehörigen, Freunden, Nachbarn oder zum Bekanntenkreis beeinträchtigt

(Bitte kreuzen Sie nur eine Zahl an)

Überhaupt nicht **. . . . . . . . . . . . . . . . . . . . . . .**1

Etwas **. . . . . . . . . . . . . . . . . . . . . . . . . . . . . . .**2

Mäßig **. . . . . . . . . . . . . . . . . . . . . . . . . . . . . . .**3

Ziemlich **. . . . . . . . . . . . . . . . . . . . . . . . . . . . .**4

Sehr **. . . . . . . . . . . . . . . . . . . . . . . . . . . . . . . .**5

**7.** Wie stark waren Ihre Schmerzen **in den vergangenen 4 Wochen**?

(Bitte kreuzen Sie nur eine Zahl an)

Ich hatte keine Schmerzen **. . . . . . . . . . . . . .**1

Sehr leicht **. . . . . . . . . . . . . . . . . . . . . . . . . . .**2

Leicht **. . . . . . . . . . . . . . . . . . . . . . . . . . . . . . .**3

Mäßig **. . . . . . . . . . . . . . . . . . . . . . . . . . . . . . .**4

Stark **. . . . . . . . . . . . . . . . . . . . . . . . . . . . . . . .**5

Sehr stark **. . . . . . . . . . . . . . . . . . . . . . . . . . . .**6

**8.** Inwieweit haben die Schmerzen Sie **in den vergangenen 4 Wochen** bei der Ausübung Ihrer Alltagstätigkeiten zu Hause und im Beruf behindert

(Bitte kreuzen Sie nur eine Zahl an)

Überhaupt nicht **. . . . . . . . . . . . . . . . . . . . . . .**1

Ein bisschen **. . . . . . . . . . . . . . . . . . . . . . . . . .**2

Mäßig **. . . . . . . . . . . . . . . . . . . . . . . . . . . . . . .**3

Ziemlich **. . . . . . . . . . . . . . . . . . . . . . . . . . . . .**4

Sehr **. . . . . . . . . . . . . . . . . . . . . . . . . . . . . . . .**5

**9.** In diesen Fragen geht es darum, wie Sie sich fühlen und wie es Ihnen **in den vergangenen 4 Wochen** gegangen ist. (Bitte kreuzen Sie in jeder Zeile die Zahl an, die Ihrem Befinden am ehesten entspricht). Wie oft waren Sie in den vergangenen **4 Wochen**...**.**

(Bitte kreuzen Sie in jeder Zeile nur eine Zahl an)

| **Befinden** | **immer** | **Mei-**  **stens** | **Ziemlich**  **oft** | **Manch-**  **mal** | **Selten** | **Nie** |
| --- | --- | --- | --- | --- | --- | --- |
| a. ...voller Schwung? | 1 | 2 | 3 | 4 | 5 | 6 |
| b. ...sehr nervös? | 1 | 2 | 3 | 4 | 5 | 6 |
| c. ...so niedergeschlagen,  daß Sie nichts aufheitern  konnte? | 1 | 2 | 3 | 4 | 5 | 6 |
| d. ...so ruhig und gelassen? | 1 | 2 | 3 | 4 | 5 | 6 |
| e. ...voller Energie? | 1 | 2 | 3 | 4 | 5 | 6 |
| f. ...entmutigt und traurig? | 1 | 2 | 3 | 4 | 5 | 6 |
| g. ...erschöpft? | 1 | 2 | 3 | 4 | 5 | 6 |
| h. ...glücklich? | 1 | 2 | 3 | 4 | 5 | 6 |
| i. ...müde? | 1 | 2 | 3 | 4 | 5 | 6 |

**10.** Wie häufig haben Ihre körperliche Gesundheit oder seelischen Probleme **in den**

**vergangenen 4 Wochen** Ihre Kontakte zu anderen Menschen (Besuche bei

Freunden, Verwandten usw.) beeinträchtigt?

(Bitte kreuzen Sie nur eine Zahl an)

Immer **. . . . . . . . . . . . . . . . . . . . . . . . . . . . . . . . . .**1

Meistens **. . . . . . . . . . . . . . . . . . . . . . . . . . . . . . . .**2

Manchmal **. . . . . . . . . . . . . . . . . . . . . . . . . . . . . . .**3

Selten **. . . . . . . . . . . . . . . . . . . . . . . . . . . . . . . . . .**4

Nie **. . . . . . . . . . . . . . . . . . . . . . . . . . . . . . . . . . . . .**5

**11.** Inwieweit trifft jede der folgenden Aussagen auf Sie zu?

(Bitte kreuzen Sie in jeder Zeile nur eine Zahl an)

| **AUSSAGEN** | **Trifft**  **ganz**  **zu** | **Trifft**  **weitge-**  **hend**  **zu** | **Weiß**  **nicht** | **Trifft**  **weitge-**  **hend**  **nicht zu** | **Trifft**  **über-**  **haupt**  **nicht zu** |
| --- | --- | --- | --- | --- | --- |
| a. Ich scheine etwas leichter als  andere krank zu werden | 1 | 2 | 3 | 4 | 5 |
| b. Ich bin genauso gesund wie alle  anderen, die ich kenne | 1 | 2 | 3 | 4 | 5 |
| c. Ich erwarte, daß meine  Gesundheit nachlässt | 1 | 2 | 3 | 4 | 5 |
| d. Ich erfreue mich ausgezeichneter  Gesundheit | 1 | 2 | 3 | 4 | 5 |

**6´- Walk - Test**

**Patient:**

Begleitperson:

Datum der Untersuchung:

Uhrzeit:

Medikamenteneinnahme: ja / nein

Vor dem Test: Nach dem Test:

Blutdruck: Blutdruck:

Herzfrequenz: Herzfrequenz:

**m**

**Erzielte Meter:**

Vorzeitiger Abbruch: ja / nein

Abbruchzeit:

Abbruchgrund:

Bemerkungen / Sonstiges:

**Therapieplan Gruppe A: Konventionelle Therapie**

|  | Montag | Dienstag | Mittwoch | Donnerstag | Freitag | Samstag | Sonntag |
| --- | --- | --- | --- | --- | --- | --- | --- |
| Uhrzeit |  |  |  |  |  |  |  |
|  |  |  |  |  |  |  |  |
| 7.30 - 8.00 |  |  |  |  |  |  |  |
| 8.00 - 8.30 | Anreise | Frühstück | Frühstück | Frühstück | Frühstück |  |  |
| 8.30 - 9.00 |  | Gehtest | Arztvisite | Arztvisite | Arztvisite |  |  |
| 9.00 - 9.30 | Untersuchng. | Herzecho | Massage | Atemgynastik | Massage | Frühstück | Frühstück |
| 9.30 - 10.00 | und | Bel.-EKG |  | ( Gruppe ) |  |  |  |
| 10.00 - 10.30 | Anamnese | Spirometrie | Inhalation |  | Inhalation |  |  |
| 10.30 - 11.00 | Einführung in | Blutgase |  | Inhalation |  |  |  |
| 11.00 - 11.30 | Klin.Abläufe | Massage |  |  |  |  |  |
| 11.30 - 12.00 | Mittagessen | Mittagessen | Mittagessen | Mittagessen | Mittagessen | Mittagessen | Mittagessen |
| 12.00 - 12.30 | Mittagsruhe | Mittagsruhe | Mittagsruhe | Mittagsruhe | Mittagsruhe | Mittagsruhe | Mittagsruhe |
| 12.30 - 13.00 | Mittagsruhe | Mittagsruhe | Mittagsruhe | Mittagsruhe | Mittagsruhe | Mittagsruhe | Mittagsruhe |
| 13.00 - 13.30 | Mittagsruhe | Mittagsruhe | Mittagsruhe | Mittagsruhe | Mittagsruhe | Mittagsruhe | Mittagsruhe |
| 13.30 - 14.00 | Thor.Röntgen | Psychologe | P.M.Relax. | Psychologe | P.M.Relax. |  |  |
| 14.00 - 14.30 | EKG |  |  |  |  |  |  |
| 14.30 - 15.00 | LZ-EKG | Ernährungsberatung | Atemgynastik | | Atemgynastik | |  |
| 15.00 - 15.30 | Vortrag |  | ( Gruppe ) | Vortrag | ( Gruppe ) |  |  |
| 15.30 - 16.00 |  |  |  |  |  |  |  |
| 16.00 - 16.30 |  |  |  |  |  |  |  |
| 16.30 - 17.00 |  |  |  |  |  |  |  |
| 17.00 - 17.30 |  |  |  |  |  |  |  |
| 17.30 - 18.00 | Abendessen | Abendessen | Abendessen | Abendessen | Abendessen | Abendessen | Abendessen |
| 18.00 - 18.30 |  |  |  |  |  |  |  |
| 18.30 - 19.00 |  |  |  |  |  |  |  |

**Therapieplan Gruppe B: Atem- und Bewegungstherapie**

|  | Montag | Dienstag | Mittwoch | Donnerstag | Freitag | Samstag | Sonntag |
| --- | --- | --- | --- | --- | --- | --- | --- |
| Uhrzeit |  |  |  |  |  |  |  |
|  |  |  |  |  |  |  |  |
| 7.30 - 8.00 |  | Frühstück | Frühstück | Frühstück | Frühstück | Frühstück | Frühstück |
| 8.00 - 8.30 | Anreise | Gehtest |  | Arztvisite | Arztvisite |  |  |
| 8.30 - 9.00 |  | Arztvisite | Intervall-Train. | Intervall-Train. | Intervall-Train. | Intervall-Train. | Intervall-Train. |
| 9.00 - 9.30 | Untersuchng. | Herzecho | Ruhe | Ruhe | Ruhe | Ruhe | Ruhe |
| 9.30 - 10.00 | und | Bel.-EKG | Inhalationen | Inhalationen | Inhalationen |  |  |
| 10.00 - 10.30 | Anamnese | Spirometrie |  | Massage |  |  |  |
| 10.30 - 11.00 | Einführung in | Blutgase | Terrain-Train. |  | Terrain-Train. |  |  |
| 11.00 - 11.30 | Klin.Abläufe | Massage |  | LZ-Blutdruck |  |  |  |
| 11.30 - 12.00 | Mittagessen | Mittagessen | Mittagessen | Mittagessen | Mittagessen | Mittagessen | Mittagessen |
| 12.00 - 12.30 | Mittagsruhe | Mittagsruhe | Mittagsruhe | Mittagsruhe | Mittagsruhe | Mittagsruhe | Mittagsruhe |
| 12.30 - 13.00 | Mittagsruhe | Mittagsruhe | Mittagsruhe | Mittagsruhe | Mittagsruhe | Mittagsruhe | Mittagsruhe |
| 13.00 - 13.30 | Mittagsruhe | Mittagsruhe | Mittagsruhe | Mittagsruhe | Mittagsruhe | Mittagsruhe | Mittagsruhe |
| 13.30 - 14.00 | Thor.Röntgen | Psychologe | P.M.Relax. | Psychologe | P.M.Relax. |  |  |
| 14.00 - 14.30 | EKG |  |  |  |  |  |  |
| 14.30 - 15.00 | LZ-EKG | Atem-Traing. | Atem-Traing. | Atem-Traing. | Atem-Traing. |  |  |
| 15.00 - 15.30 |  | Gymnastik |  | Gymnastik |  |  |  |
| 15.30 - 16.00 | Mentales | Kraft-Training | Mentales | Kraft-Training | Mentales |  |  |
| 16.00 - 16.30 | Gehtraining | Vortrag | Gehtraining | Vortrag | Gehtraining |  |  |
| 16.30 - 17.00 |  |  |  |  |  |  |  |
| 17.00 - 17.30 |  |  |  |  |  |  |  |
| 17.30 - 18.00 | Abendessen | Abendessen | Abendessen | Abendessen | Abendessen | Abendessen | Abendessen |
| 18.00 - 18.30 |  |  |  |  |  |  |  |
| 18.30 - 19.00 |  |  |  |  |  |  |  |
